# Distinct roles of monosaccharides in the inhibition of the carbohydrate‐binding module and catalytic domain of Cel7A

**DOI:** 10.1002/pro.70848

**Published:** 2026-11-09

**Authors:** Ander F. Pereira, Leandro Martínez, Munir S. Skaf

**Affiliations:** ^1^ Institute of Chemistry and Center for Computing in Engineering & Science Universidade Estadual de Campinas (UNICAMP) Campinas SP Brazil

**Keywords:** Cel7A, Kirkwood–Buff theory, minimum‐distance distribution functions, sugar‐mediated inhibition

## Abstract

The enzymatic breakdown of lignocellulosic biomass is a critical step toward the sustainable production of biochemicals. The cellobiohydrolase Cel7A plays a key role in this process by hydrolyzing cellulose. Both the primary reaction product (cellobiose) and secondary monosaccharides (glucose, mannose, and xylose) inhibit Cel7A's activity by interacting with its carbohydrate‐binding module (CBM) and catalytic domain (CD). Previous works have established that while glucose directly slows the enzyme's catalytic rate (*k*_cat_), mannose and xylose primarily reduce its association rate (*k*_on_) and overall processivity. To uncover the microscopic mechanisms behind these observations, we employed all‐atom molecular dynamics simulations combined with the Kirkwood–Buff solution theory. Our results reveal an unsuspected contrast in the solvation preferences of the two domains: while sugars are preferentially excluded from the CD, the CBM exhibits preferential sugar binding. We observed a high density of sugars along the CBM's planar binding face (residues Y5, Y31, and Y32), as well as near I11, S14, S21, V27, L28, and P30. Xylose, the most potent experimental inhibitor, displays the highest density near the CBM residues comprising the cellulose‐binding face, which could interfere with the enzyme's anchoring of cellulose. Furthermore, we observed sugar accumulation at the entrance of the CD tunnel and within the tunnel‐binding loops (B2, B3, and A3), providing a structural explanation for the obstruction of the enzyme's processive cycle. By reconciling broader solvation preferences with highly specific site interactions, our findings offer a picture of how glucose and hemicellulose‐derived sugars inhibit Cel7A.

## 1 INTRODUCTION

Lignocellulosic biomass is an abundant and renewable resource for the sustainable production of biofuels and industrial bioproducts. It is primarily composed of cellulose (30%–45%), hemicellulose (20%–40%), and lignin (10%–25%), which together form a complex and highly resistant plant cell wall architecture (Faria et al., 2024; Sulis et al., 2025). Cellulose is a linear polysaccharide composed of highly stable β‐1,4‐linked D‐glucose units. It is intertwined with hemicellulose—a heterogeneous branched polymer of pentoses and hexoses, such as xylose and mannose–and lignin, a phenolic polymer. In the context of biofuel production, cellulose constitutes the principal source of fermentable sugars.

Converting crystalline cellulose into fermentable sugars—an essential step in producing renewable fuels and high‐value products—relies on a dual approach: physicochemical pretreatment paired with multi‐component enzymatic systems. Pretreatment is designed to break down the resilient intra‐ and intermolecular bonds within the lignocellulose structure, ultimately exposing the material and making it more accessible to enzymes. However, this breakdown process presents a significant challenge. The very products generated by biomass degradation, including cellobiose, glucose, mannose, xylose, and various phenolic compounds, act as known enzymatic inhibitors (Jiang et al., 2025; Nong et al., 2024; Silveira & Skaf, 2015; Zhai et al., 2016; Zhai et al., 2018). Furthermore, even after rigorous pretreatment efforts to dismantle the heterogeneous lignocellulose structure, stubborn residual components often remain adsorbed to the cellulose surface. Together, these factors create a complex, inhibitory environment that severely hinders the efficiency of the enzymatic system (Kumar et al., 2020).

Among biomass‐degrading enzymes, Cel7A is one of the main cellobiohydrolases secreted by the filamentous fungus *Trichoderma reesei* (or *Hypocrea jecorina*) (Martinez et al., 2008), typically representing more than half of the total protein secretion profile (Jeoh et al., 2008). Cel7A features a modular architecture comprised of a CBM connected with the CD by an intrinsically disordered glycosylated linker, rich in proline, serine, and threonine (Beckham et al., 2010; Divne et al., 1998). The CD exhibits a long, tunnel‐shaped substrate‐binding crevice and eight major loops (A1–A4 and B1–B4) that cover a cleft formed by a β‐sheet sandwich. The *Tr*Cel7A CD has a 50 Å‐long substrate‐binding site that binds to the cellulose chain and hydrolyzes glycosidic bonds to produce cellobiose from the reducing end of the chain (Divne et al., 1998; Haviland et al., 2021). Its CBM facilitates the adsorption of the enzyme onto the cellulose surface, mainly through the triple tyrosines (Y5, Y31, Y32), which align on the flat surface of the crystalline cellulose via hydrophobic interactions, CH–π, and π–π stacking (Tokunaga et al., 2019).

While the macroscopic impact of sugars on Cel7A inhibition has been reported using various experimental techniques (Bezerra et al., 2011; Gruno et al., 2004; Jiang et al., 2025; Murphy et al., 2013; Nong et al., 2024; Zhai et al., 2018), a molecular‐level explanation for the distinct roles of CBM and CD remains elusive. Recently, Nong et al. suggested that cellobiose reduces both the landing rate and processive movement of the enzyme, likely due to high‐affinity binding at the “front‐door” (W40) and “back‐door” (W376) sites in the CD tunnel (Nong et al., 2024). Beyond cellobiose, monosaccharides like glucose, mannose, and xylose also exert inhibitory effects (Jiang et al., 2025; Ramos & Martínez, 2025; Zhai et al., 2018). Interestingly, these monosaccharides do not all sabotage the enzyme in the same way. While glucose directly impairs the enzyme's core catalytic activity, mannose and xylose take a different route: they primarily reduce the enzyme's ability to productively bind and move along the cellulose surface (Zhai et al., 2018). This mechanistic distinction is strongly supported by recent kinetic modeling from Jiang et al. (2025). Their work demonstrated that rather than affecting the actual catalytic rate (*k*_cat_), mannose and xylose drive inhibition by effectively lowering both the enzyme's association rate (*k*_*on*_) and its overall processivity.

To uncover the microscopic mechanisms behind this inhibition, we performed molecular dynamics (MD) simulations of the enzyme (both CBM and CD domains) in various concentrations of glucose, mannose, and xylose. We characterize the solvation structures using minimum‐distance distribution functions (MDDFs) (Martínez & Shimizu, 2017) and the Kirkwood–Buff (KB) solvation theory (Shimizu & Boon, 2004). Our findings reveal a notable preference: sugars are preferentially excluded from the CD, but preferentially bound to the CBM, relative to water. This preference is particularly pronounced around a specific set of conserved tyrosine residues (Y5, Y31, and Y32) that make up the enzyme's flat, cellulose‐binding face. Historically, the CBM's susceptibility to inhibition has been underestimated because of its naturally high affinity for crystalline cellulose (Jung et al., 2013). However, our simulations suggest that interactions between these key tyrosines and the surrounding sugars—especially xylose—may alter the local thermodynamics governing initial substrate contact, providing a molecular basis for the previously observed decrease in the enzyme's landing rate (*k*_on_) (Jiang et al., 2025).

At the same time, we observed a contrasting pattern at the CD. While the broader surface of the CD generally excludes these sugars, site‐specific interactions still occur at key structural points. Specifically, we noted both specific and non‐specific interactions at the tunnel entrance and around the tunnel‐enclosing loops (B2, B3, and A3). These localized sugar interactions reveal high‐affinity sites within the catalytic tunnel that could compete with substrate binding and potentially hinder processive movement along the cellulose chain. Taken together, our findings provide a solid thermodynamic framework that connects domain‐specific solvation preferences with residue‐level structural interactions, offering a molecular rationale for how these sugars inhibit Cel7A during processive hydrolysis.

## 2 METHODS

### 2.1 Molecular dynamics simulations and analyses

We performed MD simulations for the catalytic domain (CD, PDB ID: 8CEL) (Divne et al., 1998) and the carbohydrate‐binding module (CBM, PDB ID: 2CBH) (Kraulis et al., 1989) of the *Tr*Cel7A enzyme in the presence of glucose, mannose, and xylose (independently in their α‐ and β‐anomeric forms, Figure S1). To simulate the unliganded (*apo*) CD, the cellulose chain was removed from the 8CEL structure. For each system, eight distinct concentrations ranging from 0.1 to 2.0 mol L^−1^ were simulated.

The final protein structures were generated using the CHARMM‐GUI platform (Brooks et al., 2009; Jo et al., 2008; Lee et al., 2016), reproducing the specific protonation states of the catalytic triad (Glu212 deprotonated; Asp214 protonated and Glu217 protonated) (Knott et al., 2014), the native disulfide bridges (Divne et al., 1998; Kraulis et al., 1989), and glycosylation patterns for both domains (Chen et al., 2014; Harrison et al., 1998). For the CD, 22 potassium ions were added to neutralize the system. In contrast, the CBM is neutral. Following native glycosylation profiles, Thr1 and Ser3 of the CBM were O‐glycosylated with a single mannose (Harrison et al., 1998). In the CD domain, Asn45, Asn270, and Asn384 were N‐glycosylated by Man_5_GlcNAc_2_ glycan (Hu et al., 2001).

Solutes were solvated in orthorhombic boxes using Packmol (Martínez et al., 2009; Martínez & Martínez, 2003), containing distinct concentrations of the monosaccharides. Details of each system, including the box sides and the number of solvent molecules, are shown in Tables S1 and S2. The CHARMM36m force field (Huang et al., 2017; MacKerell et al., 1998) was employed for the protein, and the TIP3P model (Jorgensen et al., 1983) was used for water. Sugar molecules were parameterized utilizing the ADD force field, which reproduces the additivity behavior of sugar–protein interaction (Arsiccio et al., 2020).

Each system underwent 2000 steps of steepest descent minimization, succeeded by two 1 ns equilibration simulations in the NVT and NPT ensembles. During equilibration, harmonic restraints of 20 and 10 kcal mol^−1^ were applied to the backbone (C, O, N, and Cα atoms) and heavy atoms of side chains, respectively. Subsequently, a 20 ns unrestrained equilibration was performed in the NPT ensemble. Production runs consisted of 20 independent replicas of 80 ns for the CBM and 5 replicas of 1000 ns (1 μs) for the CD per system.

Snapshots were saved every 20 ps (CBM) or 200 ps (CD), resulting in 4000 and 5000 frames per system, respectively, for subsequent analyses. Simulations were conducted at 300 K, under a constant pressure of 1 bar using the Parrinello–Rahman algorithm (Parrinello & Rahman, 1981), employing a relaxation time of 2 ps and an isothermal compressibility of 4.5 × 10^−5^ bar^−1^. Temperature control was achieved using a stochastic velocity‐rescaling thermostat with a 0.1 ps period (Bussi et al., 2007). Periodic boundary conditions were applied with a 1.2 nm short‐range cutoff. Long‐range electrostatics were computed via the Particle‐Mesh Ewald (PME) (Darden et al., 1993) summation method with a 0.16 nm grid spacing. Bonds involving hydrogen were constrained using the LINCS algorithm (Hess et al., 1997), and equations of motion were integrated via the leap‐frog algorithm with a 2 fs time step using GROMACS 2023 (Abraham et al., 2015; Van Der Spoel et al., 2005).

### 2.2 Structural analysis

The protein secondary structures for each system were estimated using the STRIDE algorithm (Frishman & Argos, 1995; Kabsch & Sander, 1983) via the *ss_map* function of MolSimToolkit.jl (https://github.com/m3g/MolSimToolkit.jl—v1.31.1). Local protein flexibility was assessed through Root Mean Square Fluctuation (RMSF) calculations, with structural alignment performed using the *mdlovofit* function (Martínez, 2015; Martínez et al., 2007) as implemented in the same package. Conformational changes in the B3 loop (residues G244–G253) were further characterized by calculating its root mean square deviation (RMSD) after structural alignment of the CD. The RMSD distributions were represented as kernel density estimations (KDE) using the *density* function from the StatsPlots.jl package (https://github.com/JuliaPlots/StatsPlots.jl—v0.15.8). Changes in the exposure of the CD to the solvent were quantified by the solvent accessible surface area (SASA), computed using the Shrake–Rupley algorithm as implemented in the *sasa_particles* and *sasa* functions of PDBTools.jl (Martínez & Lima, 2026; Shrake & Rupley, 1973) and MolSimToolkit.jl, with a 0.14 nm probe radius. To specifically evaluate the shielding effect of the sugars, the SASA was calculated by treating the sugar molecules as part of the excluded volume, thus measuring the protein surface area accessible to water molecules. Throughout this work, this metric is referred to as water accessible surface area (WASA). All data were processed and visualized using the Plots.jl package (Christ et al., 2023).

### 2.3 Thermodynamics of solvation

To quantify the local and global solvation patterns of Cel7A in the presence of sugars, we employed MDDFs (Martínez & Shimizu, 2017) and the KB theory (Newman, 1994) using the ComplexMixtures.jl (https://github.com/m3g/ComplexMixtures.jl—v2.18.1) package (Martínez, 2022). The MDDF calculation allows for its decomposition into atomic and group contributions from solute and solvent. Additionally, the package was used to compute density maps of solvent molecules around each protein residue (e.g., Figure 1g), the residue‐level decomposition of preferential interactions (Figure 4a–c), and the 3D grid representation of the average solvent density (Figure 5d).

**FIGURE 1:**
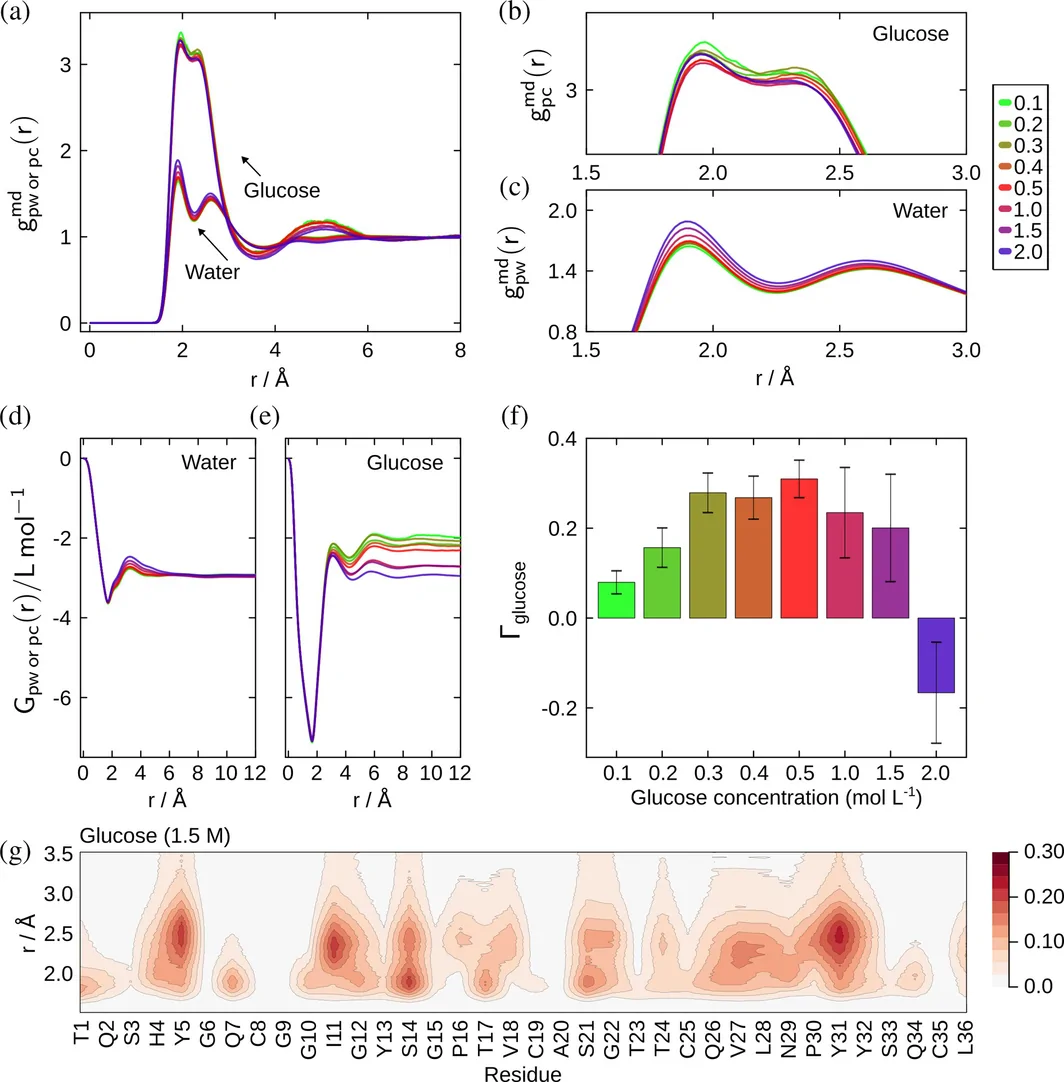
Solvation structures of the Cel7A CBM in β‐D‐glucose solutions. (a) MDDFs of water ($g_{\mathrm{pw}}^{\mathrm{md}}$) and β‐D‐glucose ($g_{\mathrm{pc}}^{\mathrm{md}}$) for the CBM at eight concentrations ranging from 0.1 (green) to 2.0 mol L^−1^ (purple). (b) and (c) are the most prominent peaks in the MDDFs of β‐D‐glucose and water. (d) and (e) show the KBIs for water ($G_{\mathrm{pw}}\left(r\right)$) and β‐D‐glucose ($G_{\mathrm{pc}}\left(r\right)$). (f) Preferential interaction parameters (*Γ*). (g) 2D MDDF per residue in the vicinity of the CBM. Red regions indicate greater β‐D‐glucose density near the CBM residues.

MDDFs describe the distribution of solvent molecules around the protein surface, providing a clear representation of solvation structures for complex solutes. Formally, the MDDF, $g_{\mathrm{ps}}^{\mathrm{md}}\left(r\right)$, is defined as the ratio between the average number density of solute–solvent distances, $n_{s}\left(r\right)$, and the density of an ideal‐gas distribution, $n_{s}^{*}\left(r\right)$:

(1) $$g_{\mathrm{ps}}^{\mathrm{md}}\left(r\right)=\frac{n_{s}\left(r\right)}{n_{s}^{*}\left(r\right)}$$

where *r* is the minimum‐distance between the solute atoms and the atoms of each solvent molecule. For clarity, we employed the following notation: w (water), p (protein), c (cosolvent), and s (water or cosolvent). The ideal distribution of minimum‐distances is obtained by generating random solvent configurations at bulk density. Detailed discussions on the theory and implementation of MDDFs are available in previous literature (Martínez, 2022; Martínez & Shimizu, 2017; Pereira et al., 2022; Pereira & Martínez, 2025; Piccoli & Martínez, 2020).

Within this framework, the Kirkwood–Buff integrals (KBIs) are defined as

(2) $$G_{\mathrm{ps}}=\frac{1}{\rho_{s}}\int_{0}^{\infty }\left[n_{s}\left(r\right)-n_{s}^{*}\left(r\right)\right]S\left(r\right)\mathrm{dr}$$

where $S\left(r\right)$ is the surface area at a minimum‐distance r from the solute, which accounts for the solute's shape. For a sufficiently large finite sub‐volume, the integral of Equation (2) can be expressed as:

(3) $$G_{\mathrm{ps}}\approx \frac{1}{\rho_{s}}\left[N_{\mathrm{ps}}\left(R\right)-N_{\mathrm{ps}}^{*}\left(R\right)\right]$$

where $N_{\mathrm{ps}}\left(R\right)$ and $N_{\mathrm{ps}}^{*}\left(R\right)$ are the number of minimum distances between the protein and the solvent within *R* in a real and ideal systems, respectively. *R* is a distance large enough to encompass the entire solvation shell affected by the presence of the solute. The KBIs are the limit of $G_{\mathrm{ps}}\left(R\right)$ for large *R*, and represent the excess (or deficit) volume occupied by the cosolvent relative to the bulk. In this study, the bulk‐density of water and sugars was calculated from the concentrations estimated in a shell between 10 and 15 Å from the protein surface. By using an open subvolume of the system to estimate the bulk density, finite‐size effects are minimized (Ganguly & van der Vegt, 2013; Simon et al., 2022).

The thermodynamics of competitive cosolvent accumulation or depletion is described by the preferential interaction parameter (*Γ*_pc_). In the infinitely dilute solute limit, the cosolvent preferential binding to the protein (*Γ*_pc_) can be approximated by site counts in a finite volume defined by *R*, with

(4) $$\Gamma_{\mathrm{pc}}\approx \rho_{c}\left[\;G_{\mathrm{pc}}\left(R\right)-G_{\mathrm{pw}}\left(R\right)\right]$$

Positive *Γ*_pc_ values indicate preferential solvation by the sugar, while negative values indicate preferential hydration.

### 2.4 Monosaccharide occupancy and residence time

The occupancy and residence time of sugars at CBM tyrosine residues on the cellulose‐binding face (Y5, Y31, Y32), as well as key tryptophan residues (W38, W40, W367, and W376) and loop B3 of the CD, were determined using the *occupancy* and *intermittent_correlation* functions in MolSimToolkit.jl (https://github.com/m3g/MolSimToolkit.jl—v2.2.2). Site occupancy was defined using a 3.0 Å minimum distance cutoff between any atom of the target residue and each sugar molecule. The decay of the resulting intermittent time‐autocorrelation function was fitted to a tri‐exponential model using the *fitexpdecay* function in EasyFit.jl (https://github.com/m3g/EasyFit.jl—v1.1.0).

## 3 RESULTS

### 3.1 Solvation properties of the Cel7A CBM in sugar solutions

We first focus on the effect of sugars and their concentrations on the solvation properties of the Cel7A CBM. Figure 1a–c shows the MDDFs of glucose and water around the CBM at eight distinct concentrations ranging from 0.1 to 2.0 mol L^−1^. While high cosolvent concentrations (up to 2.0 mol L^−1^) exceed typical industrial hydrolysis conditions (Andrić et al., 2010), extending the concentration range was essential to reveal non‐linear and saturation solvation regimes across both domains.

Two well‐defined solvation shells characterize the distribution of cosolvent (glucose) and solvent (water) molecules. These correspond to hydrogen‐bonding interactions (at ~1.95 and ~1.90 Å for glucose and water, respectively) and non‐specific and water‐mediated interactions (at ~2.49 and ~2.65 Å). An increase in glucose concentration leads to slightly higher MDDF peaks for water, whereas the MDDFs of glucose decrease, exhibiting fluctuations among concentrations.

The overall accumulation of glucose and water around the CBM is expressed by the KBIs, shown in Figure 1d,e. The data indicate that the exclusion volume of the CBM, causing the initial KBI drop, is partially compensated by a local density augmentation of water at distances 2.0 Å < *r* < 4.0 Å, which increases slightly with glucose concentration. However, at larger distances (*r* > 7.0 Å), the KBI for water becomes independent of glucose concentration, converging to approximately −3.0 L mol^−1^. In contrast, the glucose KBIs exhibit a marked dependence on cosolvent concentration. The highest affinity for the CBM is observed at the lowest concentration (0.1 M), with KBI values decreasing as concentration increases.

These trends are reflected in the preferential interaction parameters (*Γ*_glucose_, Figure 1f), which reveal that the CBM is preferentially solvated by glucose up to 1.5 mol L^−1^, with a maximum at 0.5 mol L^−1^. A transition occurs at 2.0 mol L^−1^, where the protein becomes preferentially hydrated. On the one hand, the inversion of preferential binding to preferential exclusion of glucose at high concentrations is expected, as the more affinity sites of the protein for glucose become saturated. On the other hand, the increase in glucose preferential binding at lower concentrations indicates that cooperative binding mechanisms must be present, thereby increasing the affinity of glucose relative to water even with the progressive saturation of individual high‐affinity sites.

Figure 1g provides a detailed map of glucose accumulation around each CBM residue at 1.5 mol L^−1^ (similar trends for other concentrations are provided in Figure S2). Red regions represent increased glucose density relative to the bulk, highlighting a clear preference for specific protein residues. As shown in Figure 1g, glucose interacts primarily with the aromatic residues on the cellulose‐binding face (Y5, Y31, and Y32), as well as with hydrophobic and polar residues, including I11, S14, S21, V27, L28, and P30. These interactions are primarily non‐specific, consistent with the previous study by Georgelis et al. (2012), which suggests this type of CBM (A‐CBM) typically engages in CH–π interactions with sugars.

Our results also show that solvation of the CBM by mannose and xylose follows similar trends to those observed for glucose, with some particularities. First, the MDDFs of both mannose and xylose also show two solvation shells, which characterize both hydrogen‐bonding and non‐specific interactions with the CBM. Nevertheless, compared with that of glucose, their densities at the first solvation shells are smaller, while the densities at the second solvation shells are greater across all sugar concentrations (Figures 2a–c, S3a–c and S4.1a–c). The maximum increase in the second solvation shell is observed for xylose (Figure 2c).

**FIGURE 2:**
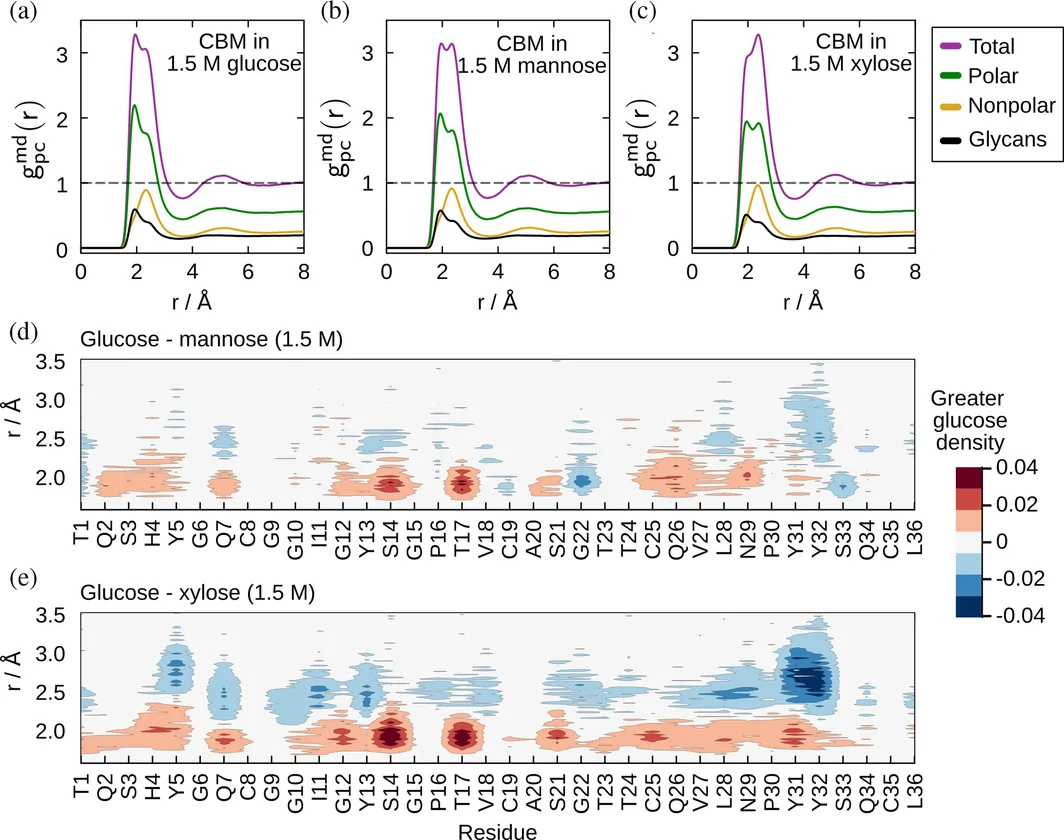
Decomposition of the total MDDFs (purple) of (a) β‐D‐glucose, (b) β‐D‐mannose, and (c) β‐D‐xylose relative to the CBM in polar (green), nonpolar (yellow), and glycans (black) contributions. (d) and (e) Differential density map of the MDDF breakdown per residue of the CBM. Red indicates greater β‐D‐glucose density and blue greater β‐D‐mannose or β‐D‐xylose density around the CBM, respectively.

A breakdown of the total MDDF into polar and nonpolar residue contributions reveals that polar residues provide the largest absolute contribution to total CBM solvation for all three sugars. This dominant polar component arises primarily from an extensive hydrogen‐bonding network between the sugar hydroxyl groups and the hydrophilic protein surface, with notable contributions from the amphipathic tyrosine residues, which are classified as polar due to their phenolic hydroxyl (–OH) group. However, while the polar contacts dominate overall solvation, non‐specific interactions between sugars and both polar and nonpolar residues increase from glucose to xylose (Figure S5). This trend is particularly evident at *r* ~ 2.5 Å in the tyrosine MDDFs (Figure S5C), indicating that the enhanced solvation of xylose is driven by its higher propensity to engage in hydrophobic interactions with the aromatic triad compared to glucose and mannose.

The primary interaction sites for mannose and xylose (Figures S6 and S7) closely match those identified for glucose (Figure 1g), indicating a conserved pattern of sugar accumulation across the CBM surface. Nevertheless, the differential density maps (Figure 2d,e) reveal quantitative differences in local sugar concentration. Mannose and, in particular, xylose demonstrate increased accumulation within the second solvation shell of the aromatic triad (Y5, Y31, and Y32) and the adjacent hydrophobic residues (I11, V27, L28, and P30). This effect is most pronounced for xylose, which displays the highest local density among the three cosolvents. This trend parallels the relative hydrophobicity of the three sugars as quantified by HPLC retention (capacity factors *K*_c_ = 0.030, 0.026, and 0.016 for xylose, mannose, and glucose, respectively; xylose > mannose > glucose) (Buttersack, 2017). Structurally, this is consistent with xylose lacking the C6 hydroxymethyl group present in the aldohexoses glucose and mannose.

The KBIs for glucose and mannose are remarkably similar, both exhibiting a transition toward preferential hydration at 2.0 mol L^−1^ (Figures 1d–f and S3.1d–f). In contrast, the KBIs for xylose remain consistently higher (Figure S4.1e), corroborating its stronger affinity for the CBM surface. Notably, while *Γ*_xylose_ decreases at 2.0 mol L^−1^, it remains positive (Figures 3 and S4.1f and Table S3), indicating that xylose resists the transition to preferential hydration observed for glucose and mannose.

**FIGURE 3:**
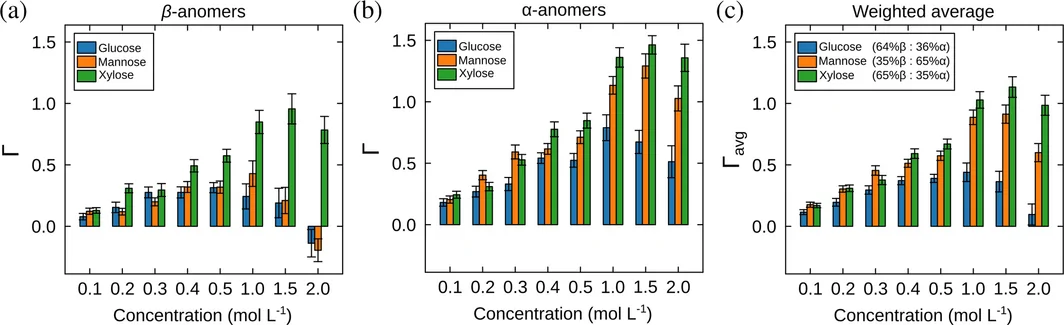
Preferential interaction parameters (*Γ*) as a function of sugar concentration for (a) β‐anomers, (b) α‐anomers, and (c) ensemble‐weighted averages (*Γ*_avg_). Data in (c) represent the weighted average of α‐ and β‐anomers according to their thermodynamic equilibrium populations in aqueous solution: D‐glucose (64% β: 36% α), D‐mannose (35% β: 65% α), and D‐xylose (65% β: 35% α). Bars indicate the mean values, and error bars represent the propagated standard error of the mean calculated across 20 independent simulation replicas.

The β form, discussed so far, is the thermodynamically predominant anomer for D‐glucose and D‐xylose in aqueous solution, representing slightly more than 60% at equilibrium (Dumouilla & Dussap, 2021; Liu et al., 2010; Schmidt et al., 1996). Conversely, the α form predominates for mannose (~65% in aqueous solution) (Dumouilla & Dussap, 2021). To account for this mutarotation equilibrium, we also simulated solutions of the α‐anomers. The α‐anomers display higher overall preferential interaction parameters (Figure 3a,b and Table S3), although the anomeric forms exhibit similar trends across concentrations, with preferential solvation reaching a maximum at intermediate concentrations and decreasing at 2.0 M. Assuming the additivity of these preferential accumulations, Figure 3c shows the weighted average of the preferential interaction parameters (*Γ*_avg_) from 0.1 to 2.0 M based on their equilibrium populations in aqueous solution (Dumouilla & Dussap, 2021; Liu et al., 2010; Schmidt et al., 1996). The relative ordering of the preferential interaction parameters is strictly conserved regardless of the sugar anomeric configuration (xylose > mannose > glucose). Crucially, direct simulations carried out with explicit anomeric mixtures at the highest concentration (2.0 M) yielded preferential solvation parameters closely matching these weighted values (Table S4), validating the additivity assumption across the studied concentration range.

The preferential interaction parameters can be decomposed into group contributions assuming that each atom is responsible for structuring the solution closer to it, in a Voronoi decomposition of the space surrounding the solute (Zheng et al., 2016). A residue‐by‐residue analysis of the contributions to *Γ* clarifies why β‐D‐xylose avoids the transition to preferential hydration. As shown in Figure 4, β‐D‐xylose not only interacts preferentially with a larger number of residues compared to β‐D‐glucose and β‐D‐mannose, but these interactions are also distinctly stronger. Remarkably, the primary individual contributor to preferential accumulation for all three sugars is invariably an aromatic residue belonging to the cellulose‐binding face (Y5, Y31, or Y32). Furthermore, the remaining two tyrosines of this binding triad consistently rank among the top contributors across the entire protein surface. β‐D‐xylose is uniquely distinguished by more widespread positive contributions attributed, by the spatial decomposition of *Γ*, to the residues that are nearby the side‐chains that display augmented interactions. The wider reach of preferential interactions maintains a positive overall *Γ*, counteracting the shift toward preferential hydration observed for β‐D‐glucose and β‐D‐mannose.

**FIGURE 4:**
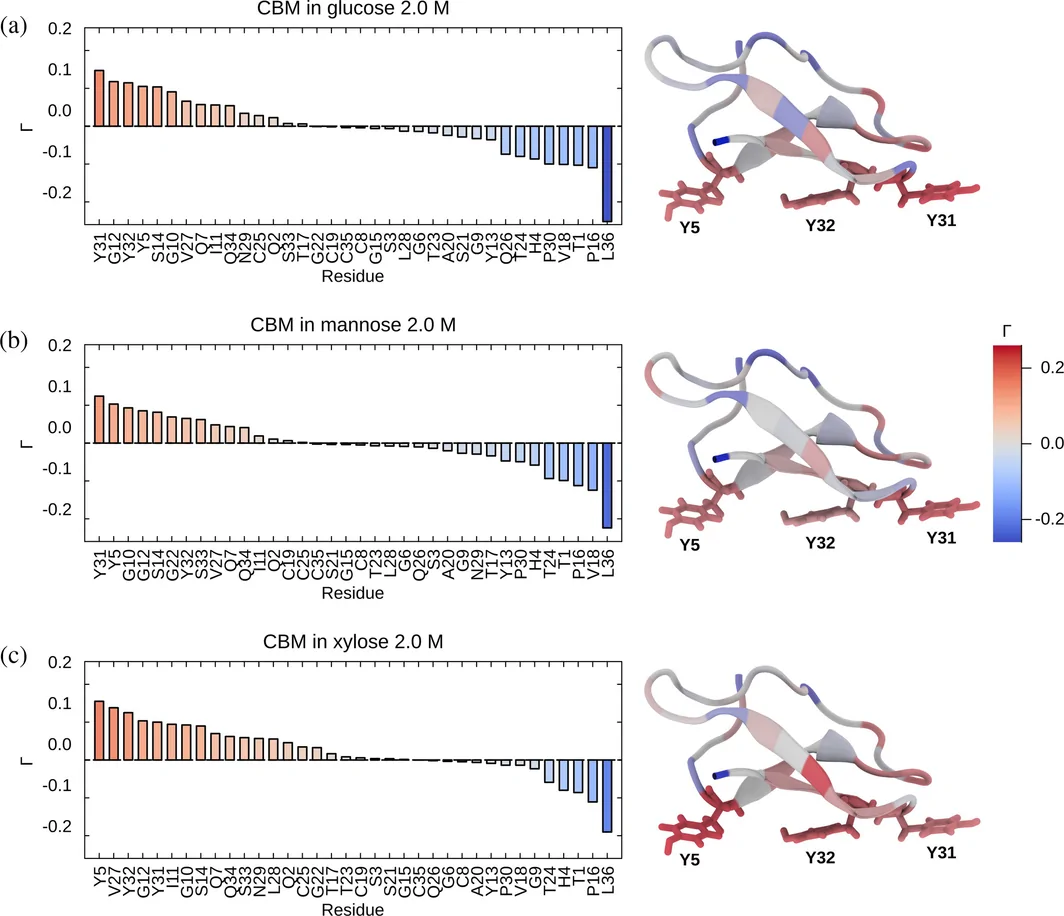
Residue‐level contributions to the preferential interaction parameters (*Γ*) for the CBM domain in 2.0 M solutions of (a) β‐D‐glucose, (b) β‐D‐mannose, and (c) β‐D‐xylose, sorted in descending order of magnitude. Positive *Γ* values (blue) indicate local preferential accumulation of the sugar with specific residues, whereas negative values (red) denote preferential exclusion. On the right, the 3D structure of the CBM domain is mapped with the corresponding color scale, highlighting that the greater contributions are found in the cellulose‐binding surface.

While preferential solvation of the CBM arises from a collective accumulation of sugars across the protein surface (Figures 1g, S2, and S6–S10), residues Y5 and Y31 exhibit the highest individual local densities of sugars at all concentrations. Moreover, the variations in MDDF peak height as a function of sugar concentration are most evident for these residues, indicating that the sugars surrounding the aromatic triad (Y5, Y31, and Y32) play a significant role in the observed preferential solvation trends.

At lower concentrations, these aromatic sites are efficiently occupied, contributing more effectively to a positive preferential interaction. At 2.0 mol L^−1^, however, while the interaction sites remain consistent (Figures S2 and S6–S10), the relative density of sugars around these residues decreases, indicating partial saturation of the sites. This is evidenced by the reduced peak intensities in the MDDFs (Figures 5a–c and S11) and lower bulk‐normalized occupancies (Tables S5 and S6 and Figure S12), which show an overall reduction from 0.5 to 2.0 mol L^−1^ across all studied sugars.

**FIGURE 5:**
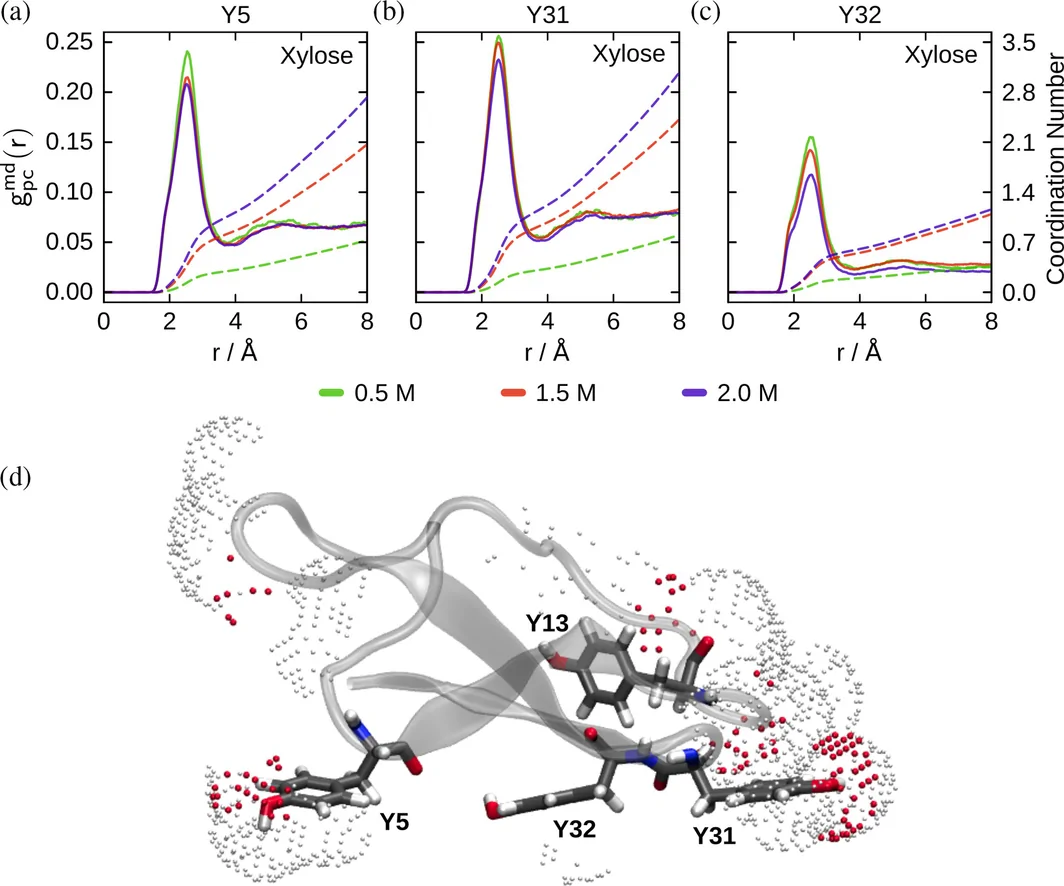
MDDF contributions for the side chains of the residues (a) Y5, (b) Y31, and (c) Y32 across different xylose concentrations. MDDFs are shown for concentrations of 0.5 mol L^−1^ (red), 1.5 mol L^−1^ (magenta), and 2.0 mol L^−1^ (purple). (d) Structure of Cel7A CBM. Tyrosine residues at the cellulose‐binding face (Y5, Y32, Y31) and Y13 are shown as licorice. Points in space indicate the regions of greater β‐D‐xylose accumulation around the CBM, colored by the atom type of the closest solute atom.

This relative depletion at high concentrations contributes to the transition toward preferential hydration for glucose and mannose, and the decrease in the preferential solvation by xylose. This effect is particularly evident for residue Y32 and is highlighted by the xylose coordination numbers in Figure 5a–c (right *y*‐axis). Unlike the variations seen in Y5 and Y31, the coordination numbers of xylose at Y32 are remarkably similar at 1.5 and 2.0 mol L^−1^, indicating saturation.

Notably, sugar density remains low around Y13 (Figures 1g and S11d–f), a residue that does not belong to the conserved binding face (Y5, Y31, and Y32). Because these three core tyrosines are essential for mediating CH–π stacking interactions with the crystalline cellulose surface, their persistent occupation by free sugar molecules likely masks the CBM's primary binding interface. This competitive masking offers a plausible explanation for why enzyme inhibition is more pronounced on crystalline cellulose than on amorphous substrates (Jiang et al., 2025). During the degradation of crystalline cellulose, enzyme efficiency relies heavily on CBM‐mediated adsorption to overcome the substrate's limited accessibility. When free sugars occupy the aromatic triad, they appear to hinder the CBM's ability to anchor the enzyme to the flat crystalline surface. Conversely, on amorphous cellulose, the catalytic domain can more readily access disordered chains, rendering the overall hydrolytic rate less dependent on the CBM's surface‐binding capacity.

### 3.2 Structural properties of the Cel7A CD in water and sugar solutions

While sugar accumulation at the CBM provides a plausible molecular basis for the experimentally observed reduction in binding to crystalline cellulose, Cel7A inhibition ultimately operates as a multi‐domain process. To explore this broader effect, we next examined how the presence of free sugars influences the structural and solvation properties of the CD. In contrast to the CBM's relatively flat binding surface, the CD features a complex, tunnel‐like architecture. This structure is enclosed by a series of flexible loops—specifically the A‐loops (A1–A4) and B‐loops (B1–B4)—that collectively form the roof of the catalytic tunnel (Figure 6a) (Momeni et al., 2013).

**FIGURE 6:**
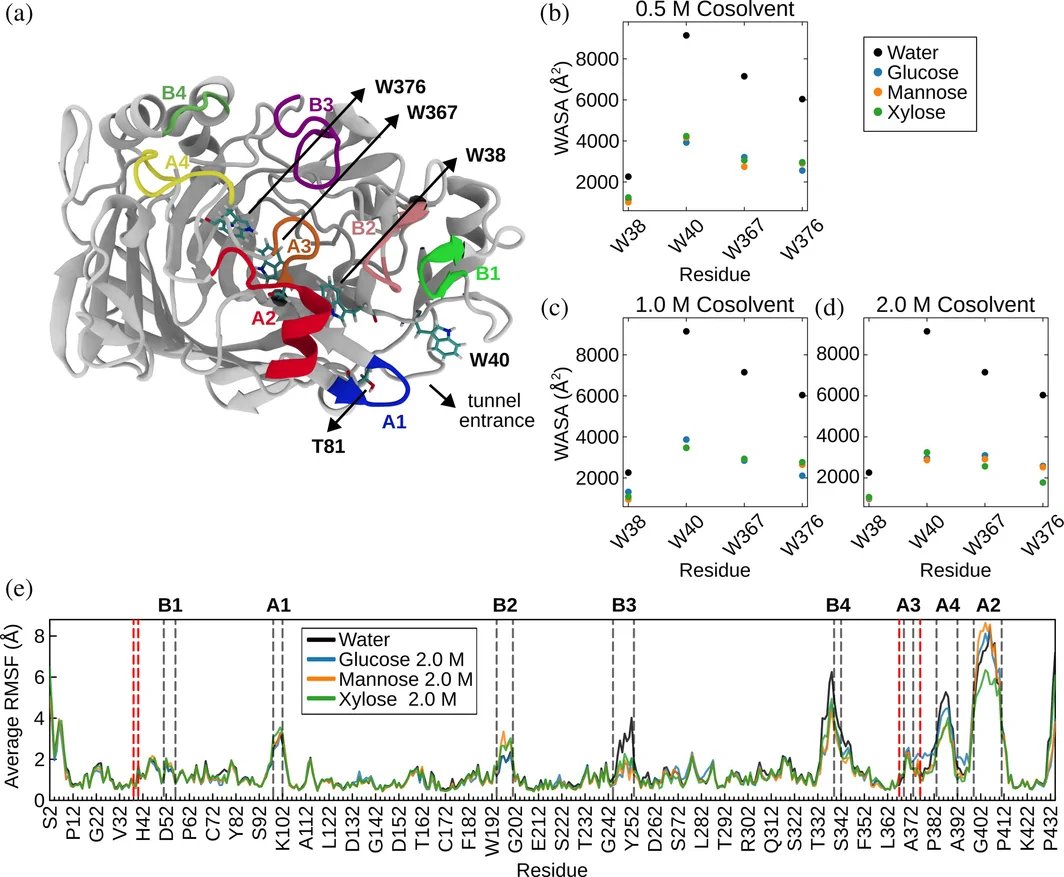
(a) Structure of Cel7A CD. A‐loops (A1–A4) are highlighted in blue, red, orange, and yellow, respectively. B‐loops (B1–B4) are highlighted in green, light pink, purple, and lime. Tryptophan residues (W40, W38, W367, and W376) and T81 are shown as licorice. (b)–(d) WASA for tryptophan residues within the catalytic tunnel in water (black) and at (b) 0.5 mol L^−1^, (c) 1.0 mol L^−1^, and (d) 2.0 mol L^−1^ of β‐D‐glucose (blue), β‐D‐mannose (orange), and β‐D‐xylose (green). (e) RMSF of the Cel7A CD in water (black line) and 1 mol L^−1^ sugar solutions (colored as above). Gray dotted lines delimit the loop regions, while red dotted lines indicate the positions of W38, W40, W367, and W376.

In its substrate‐free state (*apo*‐CD), the enzyme's A and B loops, along with its N‐ and C‐terminal regions, exhibit natural flexibility. This inherent mobility is captured by high RMSFs (Figure 6e)—most notably across loops A1, A2, A4, and B4, with only A2 and B1 loops maintaining discernible secondary structure (Figures S14 and S15). Positioned at the base of the CD near the tunnel's entrance, loop A1, alongside loop B2, is widely considered critical for overall Cel7A activity (Brunecky et al., 2024). Interestingly, specific modifications to these regions—such as the Q101A mutation in the A1 loop and the N197A/N200A double mutation in the B2 loop—actually yield higher turnover numbers than the wild‐type (WT) enzyme. However, this enhanced catalytic rate involves a structural trade‐off, as the mutations in the B2 loop simultaneously weaken the enzyme's binding affinity for crystalline cellulose (Sørensen et al., 2017).

Our simulations reveal that the presence of sugars alters loop dynamics. The A2 loop (residues Q98 to K102) exhibits a localized α‐helical content that is typically stabilized by sugar solutions compared to pure water (Figures 6e and S14). Similarly, the B3 loop (G244–G253), which is disordered and near the product‐binding site, shows a systematic reduction in RMSF in the presence of sugars at all concentrations (Figures 6e and S16). This is further supported by the distribution of the B3 loop RMSD (Figures 7a and S17), where sugar solutions shift the distribution toward lower RMSDs compared to the broader distribution observed in pure water. The suppression of these fluctuations directly impacts the dynamics of the enzyme. Previous principal component analysis (PCA) identified that the *apo*‐CD performs concerted ‘breathing’ motions, characterized by open‐close fluctuations of the tunnel‐enclosing loops that are essential for substrate processing (Silveira & Skaf, 2018), which can be suppressed by the smaller mobility of the loops in the presence of sugars.

The B3 loop is important for maintaining the high processivity of *Tr*Cel7A on crystalline cellulose by stabilizing the reducing end of the cellulose chain at the product binding subsites (+1/+2) following catalysis (von Ossowski et al., 2003). The sugar‐induced restriction of B3 flexibility could potentially modulate product release or the forward progression of the cellulose chain. Despite these localized dynamic shifts, the overall fold of the CD remains unaffected by the presence of sugars, as evidenced by the constant β‐sheet content across all concentrations (Figure S15).

Within the substrate‐binding tunnel of the *Tr*Cel7A CD, four conserved tryptophan residues (W40, W38, W367, and W376) facilitate processive hydrolysis by providing aromatic stacking interactions at the −7 (W40), −4 (W38), −2 (W367), and +1 (W376) subsites (Nakamura et al., 2013; Taylor et al., 2013). While we observed a systemic reduction in water accessibility (WASA) across nearly the entire enzyme surface upon sugar addition (Figure S18), the impact on these specific aromatic residues is particularly significant for catalysis. Even at low sugar concentrations, the WASA of these tryptophans decreases by more than 50% (Figure 6b–d), although their structural mobility remains largely unchanged (red dotted line in Figure 6e). This substantial reduction suggests that the sugars effectively shield the aromatic residues from hydration. Consequently, this masking effect is consistent with a reduced capacity for hydrophobic stacking with the cellulose chain, offering a molecular rationale for impaired substrate engagement.

### 3.3 Solvation properties of the Cel7A CD in sugar solutions

As illustrated in Figure 8, the MDDFs for the sugars (Figure 8a,c,e) and water (Figure 8b,d,f) in the CD are similar to those observed for the CBM. Among the cosolvents, glucose demonstrates the highest propensity for hydrogen bonding with the protein surface (indicated by the peak at ~1.9 Å), followed by mannose and xylose. Interestingly, the density of sugars participating in non‐specific interactions (*r* ~ 2.35 Å) remains lower than that of the first solvation shell, even in xylose solutions. The xylose MDDF peaks exhibit a modest variation. At 2.0 mol L^−1^, the xylose MDDF in the first solvation shell (*r* ~ 1.95 Å, Figure 6e) shows a slight increase compared to 0.5 and 1.5 mol L^−1^, while maintaining the lowest values in the non‐specific interaction region (*r* ~ 2.35 Å, Figure 8e).

We mapped the specific contributions of the tunnel residues (those within 5 Å of the substrate path) to the sugar distributions (Figures 8g and S19–S21). The sugars preferentially accumulate at the tunnel entrance (involving residues Q7, W40, S47, N49, D52, G53, T81, A100, Q101, K102, and V104) and around the flexible loops that enclose the tunnel—specifically B2 (N200), B3 (R251, Y252), and A3 (Y370). In contrast, sugar density remains notably low around the core catalytic triad (E212, D214, and E217) and the internal aromatic residues (W38, W367, and W376).

A notable observation is the distinct solvation of the B3 loop—particularly around residues R251 and Y252—a region essential for product coordination and processive movement (Payne et al., 2015; Ubhayasekera et al., 2005; von Ossowski et al., 2003). The localized accumulation of sugars in this area (Figures S13–S15) offers a clear molecular rationale for the structural stabilization, or reduced RMSF, discussed in the previous section. By interacting with these inherently flexible residues (Figures 8g and S19–S21), the sugars constrain the dynamic fluctuations that the enzyme requires to advance along the cellulose chain (Figure 7b).

**FIGURE 7:**
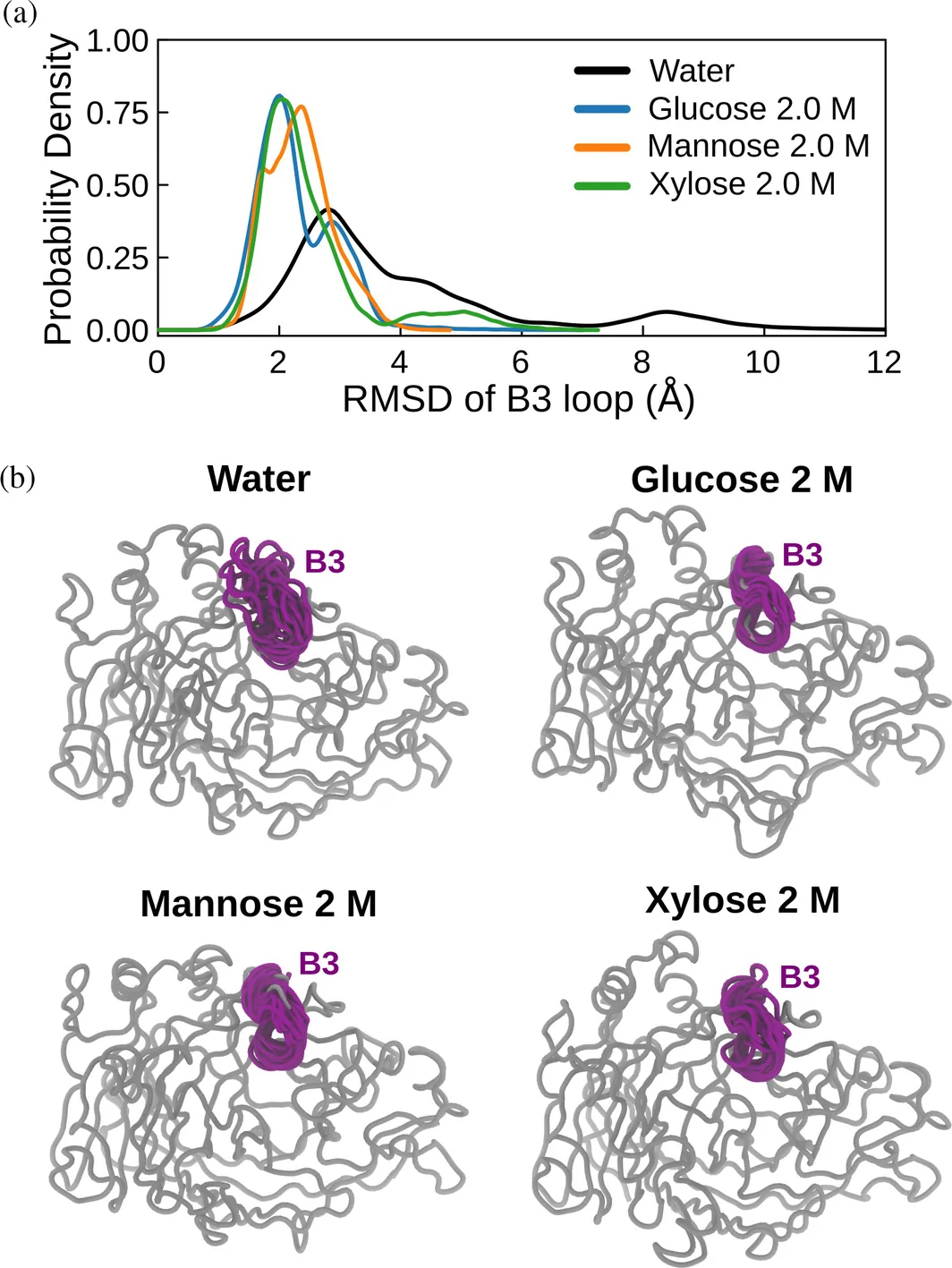
Structural stability of the B3 loop in water and sugar solutions. (a) In pure water (black) and 2.0 mol L^−1^ β‐D‐glucose (blue), β‐D‐mannose (orange), and β‐D‐xylose (green) solutions. (b) Representative conformational ensembles in water and 2.0 mol L^−1^ sugar solutions, aligned to the CD (gray) to highlight local variability. The B3 loop (purple) exhibits broader conformational sampling in water, consistent with the wider RMSD distributions shown in (a).

Differences in the density maps (Figures 8h,i, S22 and S23) further indicate that glucose displays a markedly higher preference for the catalytic acid/base residue (E217) compared to mannose or xylose. This observation supports the behavioral distinction proposed by Zhai et al. (2018), offering a structural rationale for why glucose primarily impacts catalytic activity, whereas mannose and xylose inhibit overall hydrolysis by reducing enzyme processivity. Additionally, the tendency of mannose and xylose to accumulate near W40 suggests favorable CH–π and hydrophobic interactions at the tunnel entrance. Because W40 plays a central role in substrate recognition, its occupation by these monosaccharides (Figure S24) may impede the initial binding and subsequent passage of the cellulose chain into the catalytic tunnel.

**FIGURE 8:**
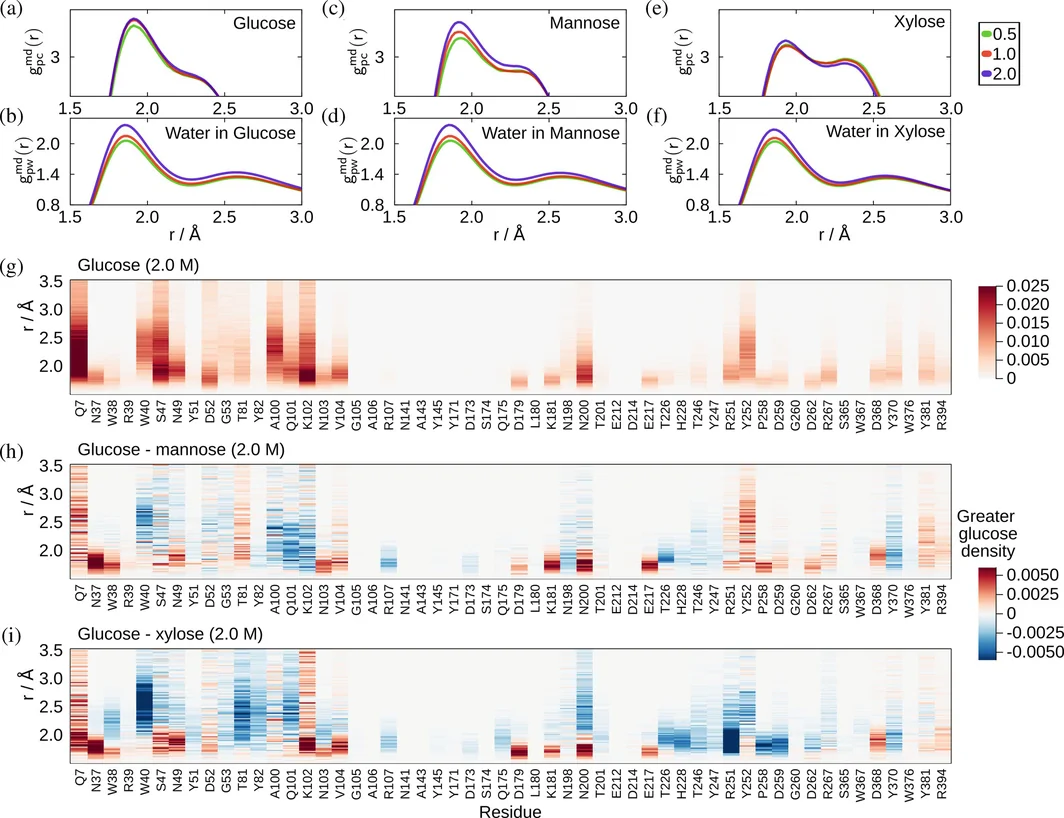
Primary MDDF peaks for (a) β‐D‐glucose, (c) β‐D‐mannose, and (e) β‐D‐xylose, respectively, with (b, d, f) showing the corresponding water MDDFs. (g) 2D MDDF maps per residue in the vicinity of the CD. Red regions indicate greater glucose density relative to the protein surface. (h) and (i) Differential MDDF maps per residue of the CD tunnel residues, where red represents higher β‐D‐glucose density, whereas blue indicates higher (h) β‐D‐mannose or (i) β‐D‐xylose density.

Figure 8i also highlights the higher xylose density around T81, which is positioned at the entrance of the catalytic domain (Figure 6a) and coordinates the substrate via hydrogen bonding. Our data suggest that the persistent occupancy of T81 by xylose could disrupt these initial polar interactions, providing a further potential mechanism for reduced processivity.

### 3.4 Domain and sugar dependence on the solvation

Figure 9 illustrates a domain‐dependent solvation response to increasing cosolvent concentrations. For the CBM, water KBIs remain largely invariant across different sugar types and concentrations (Figure 9a). In contrast, the CD demonstrates a slight but progressive increase in water KBIs under the same conditions (Figure 9d). Alongside these hydration trends, the sugar KBIs for both domains (Figure 9b,e) decrease as sugar concentration rises. Throughout these variations, xylose consistently maintains the highest affinity for both domains, as reflected by its greater KBI values.

**FIGURE 9:**
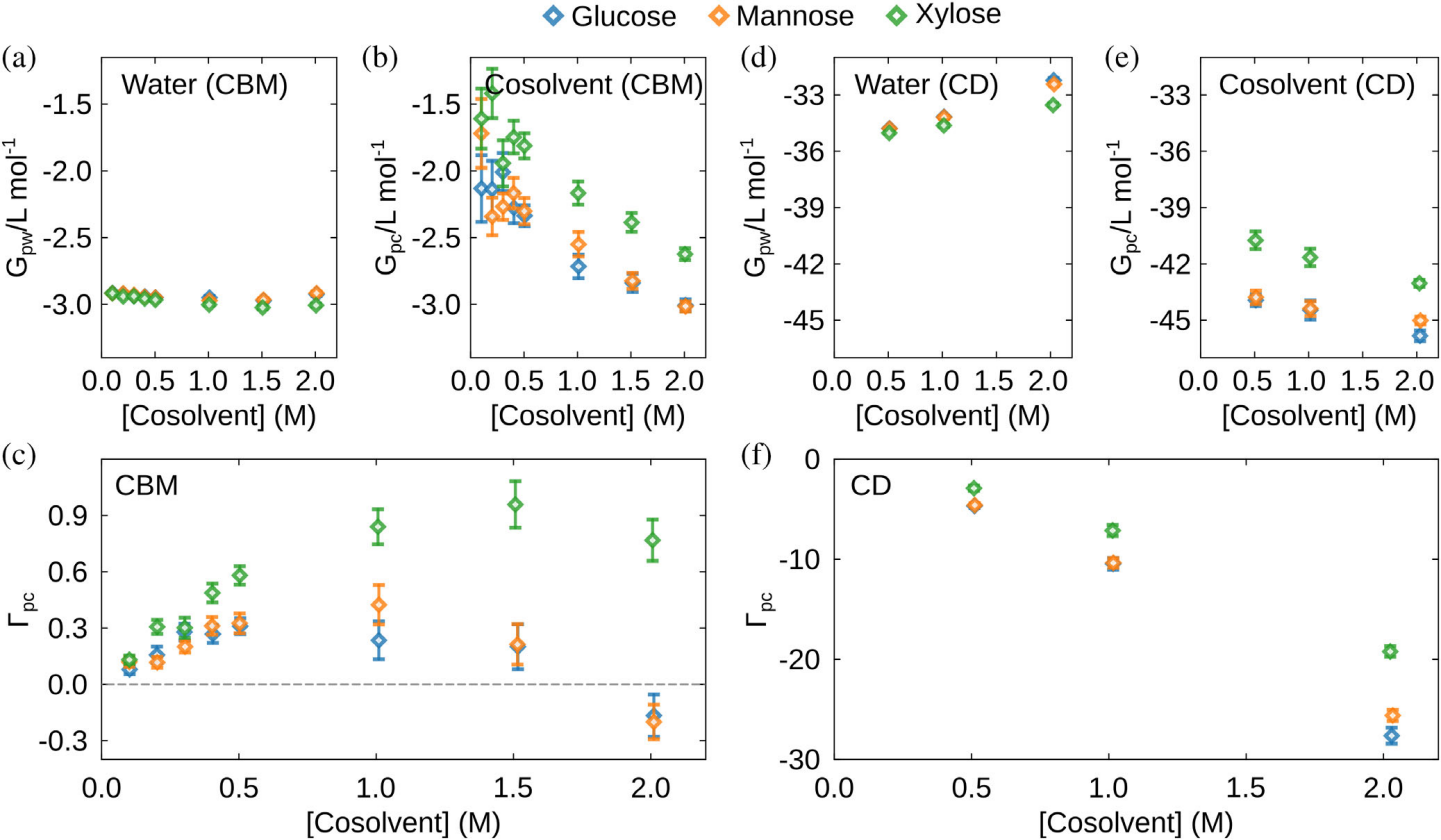
Kirkwood–Buff solvation analysis for the CBM and CD as a function of sugar concentration. (a, d) KBIs for water in β‐D‐glucose (blue), β‐D‐mannose (orange), and β‐D‐xylose (green) solutions for the (a) CBM and (d) CD. (b, e) Corresponding KB integrals for the sugars. (c, f) Preferential interaction parameters (*Γ*) highlighting the transition between preferential sugar accumulation in the CBM and preferential exclusion in the CD. Note the differences in magnitude of the preferential interactions of the two domains.

The variations in KBI magnitude between the CBM and CD reflect distinct regimes of preferential interaction (*Γ*), a trend that holds regardless of the sugar anomeric configuration (β or α) (Table S3). As detailed in Table S3 and Figure 9c,f, the CBM exhibits preferential solvation by sugars (*Γ* > 0) across most concentrations. This effect is most pronounced for xylose, which reaches a peak value of 0.96 ± 0.1 at 1.5 mol L^−1^. Conversely, the CD consistently yields negative *Γ* values, dropping to −27.6 ± 0.8 at the highest concentration. This general exclusion from the CD, juxtaposed with the accumulation at the CBM, suggests that the two domains mediate inhibition via separate mechanisms. At the CBM, sugars accumulate primarily through aromatic–carbohydrate interactions with tyrosine residues along the cellulose‐binding face. Meanwhile, at the CD, enzymatic efficiency may be modulated by localized sugar interactions near the tunnel entrance and along loops B2, B3, and A3 as discussed in the previous section.

The higher preferential solvation at the CBM tyrosine residues observed in our simulations correlates with existing experimental data showing that xylose acts as a more potent inhibitor of Cel7A productive binding and processivity than mannose (Jiang et al., 2025; Zhai et al., 2018). By more effectively occupying these key aromatic sites, xylose accumulation is consistent with a reduced availability of the CBM for anchoring onto the crystalline cellulose surface, offering a structural explanation for its greater impact on productive binding relative to mannose.

Importantly, while *Tr*Cel7A functions as a modular unit where the CBM, glycosylated linker, and catalytic domain act together during cellulose degradation, simulating the CBM and CD independently was a strategy that allowed us to deconvolve their opposing thermodynamic profiles without the effects of interdomain steric constraints.

## 4 CONCLUSION

Developing more efficient enzymatic cocktails to mitigate product inhibition during lignocellulose depolymerization requires a precise, molecular‐level understanding of inhibitor interactions. Our simulations reveal distinct differences in how glucose and hemicellulosic sugars (mannose and xylose) interact with the two primary functional domains of Cel7A. Specifically, while the CBM is preferentially solvated by these sugars—driven largely by non‐specific interactions and localized accumulation at the aromatic tyrosines of the cellulose‐binding face, the CD remains preferentially hydrated across the studied concentration range.

This competitive masking of the CBM's binding interface is consistent with a reduced availability of the cellulose‐binding face for anchoring, offering a structural explanation for previously reported reductions in the association rate *k*_on_ (Jiang et al., 2025). Furthermore, although the broader CD surface generally excludes sugars, we identified key, localized interactions at the tunnel entrance and within the tunnel‐enclosing loops (specifically B2, B3, and A3). The resulting structural stabilization and sugar accumulation, particularly within the B3 loop near the catalytic exit, provide a molecular mechanism for the observed decrease in overall Cel7A processivity.

## AUTHOR CONTRIBUTIONS

**Ander F. Pereira:** Investigation; writing – original draft; validation; formal analysis; conceptualization; methodology; writing – review and editing; data curation. **Leandro Martínez:** Conceptualization; investigation; funding acquisition; methodology; validation; formal analysis; project administration; data curation; supervision; resources; software; writing – review and editing. **Munir S. Skaf:** Conceptualization; funding acquisition; writing – review and editing; validation; methodology; project administration; supervision; resources; formal analysis.

## Supporting information


**Table S1:** System composition and simulation box parameters for the Cel7A CBM in the presence of distinct concentrations (0.1–2.0 M) of sugars (glucose, mannose, and xylose). Each system consists of 20 independent production replicates of 80 ns each, totaling 1.6 μs of simulation time per concentration.
**Table S2:** System composition and simulation box parameters for the Cel7A CD in water and in the presence of distinct concentrations (0.1–2.0 M) of sugars (glucose, mannose, and xylose). Each system consists of five independent production replicates of 1000 ns each, totaling 5.0 μs of simulation time per concentration.
**Table S3:** Preferential interaction parameters (*Γ*) of the CBM and CD for β‐D‐glucose (*Γ*_β‐D‐glucose_), α‐D‐glucose (*Γ*_α‐D‐glucose_), β‐D‐mannose (*Γ*_β‐D‐mannose_), α‐D‐mannose (*Γ*_α‐D‐mannose_), β‐D‐xylose (*Γ*_β‐D‐xylose_), and α‐D‐xylose (*Γ*_α‐D‐xylose_) solutions.
**Table S4:** Ensemble‐averaged preferential interaction parameters (*Γ*) of the CBM in aqueous solutions of glucose (*Γ*_Glucose_), mannose (*Γ*_Mannose_), and xylose (*Γ*_Xylose_). Values represent weighted averages based on experimental equilibrium ratios of β‐ and α‐anomers in water: 64%:36% for glucose, 35%:65%% for mannose, and 65%:35% for xylose. Errors denote standard errors.
**Table S5:** Occupancy of Y5, Y31, and Y32 around CBM at 3.0 Å across sugars (glucose, mannose, and xylose) and concentrations (0.5, 1.5, and 2.0).
**Table S6:** Normalized occupancy of Y5, Y31, and Y32 in the CBM at 3.5 Å across sugars (glucose, mannose, and xylose) and concentrations (0.5, 1.5, and 2.0). Normalized occupancy is defined as the occupancy divided by the average bulk density of the sugar in each system.
**Figure S1:** Structural representation of the sugars studied as cosolvents. Panels (a)–(c) show the chemical structures of (a) β‐D‐glucose, (b) β‐D‐mannose, and (c) β‐D‐xylose, whereas panels d–f show those of (d) α‐D‐glucose, (e) α‐D‐mannose, and (f) α‐D‐xylose.
**Figure S2:** 2D MDDF per residue for β‐D‐glucose in the vicinity of the Cel7A CBM. Panels (a)–(h) display the 2D MDDF for eight distinct β‐D‐glucose concentrations, ranging from 0.1 to 2.0 M. Red regions indicate greater β‐D‐glucose density near the CBM residues.
**Figure S3.1:** Solvation structures of the Cel7A CBM in β‐D‐mannose solutions. (a) MDDFs of water and β‐D‐mannose for the CBM at eight concentrations ranging from 0.1 M (green) to 2.0 M (purple). (b) and (c) are the most prominent peaks in the MDDFs of β‐D‐mannose and water. (d) and (e) show the KBIs for water and β‐D‐mannose. (f) Preferential interaction parameters (*Γ*).
**Figure S3.2:** Solvation structures of the Cel7A CBM in α‐D‐mannose solutions. (a) MDDFs of water and α‐D‐mannose for the CBM at eight concentrations ranging from 0.1 M (green) to 2.0 M (purple). (b) and (c) are the most prominent peaks in the MDDFs of α‐D‐mannose and water. (d) and (e) show the KBIs for water and α‐D‐mannose. (f) Preferential interaction parameters (*Γ*).
**Figure S4.1:** Solvation structures of the Cel7A CBM in β‐D‐xylose solutions. (a) MDDFs of water and β‐D‐xylose for the CBM at eight concentrations ranging from 0.1 M (green) to 2.0 M (purple). (b) and (c) are the most prominent peaks in the MDDFs of β‐D‐xylose and water. (d) and (e) show the KBIs for water and β‐D‐xylose. (f) Preferential interaction parameters (*Γ*).
**Figure S4.2:** Solvation structures of the Cel7A CBM in α‐D‐xylose solutions. (a) MDDFs of water and α‐D‐xylose for the CBM at eight concentrations ranging from 0.1 M (green) to 2.0 M (purple). (b) and (c) are the most prominent peaks in the MDDFs of α‐D‐xylose and water. (d) and (e) show the KBIs for water and α‐D‐xylose. (f) Preferential interaction parameters (*Γ*).
**Figure S5:** Decomposition of the total MDDFs of (a) β‐D‐glucose (blue), (b) β‐D‐mannose (orange), and (c) β‐D‐xylose (green) at 1.5 M relative to the CBM, showing polar, nonpolar, and tyrosine (Tyr) contributions.
**Figure S6:** 2D MDDF per residue for β‐D‐mannose in the vicinity of the Cel7A CBM. Panels (a)–(h) display the 2D MDDF for eight distinct β‐D‐mannose concentrations, ranging from 0.1 to 2.0 M. Red regions indicate greater β‐D‐mannose density near the CBM residues.
**Figure S7:** 2D MDDF per residue for β‐D‐xylose in the vicinity of the Cel7A CBM. Panels (a)–(h) display the 2D MDDF for eight distinct β‐D‐xylose concentrations, ranging from 0.1 to 2.0 M. Red regions indicate greater β‐D‐xylose density near the CBM residues.
**Figure S8:** 2D MDDF per residue for α‐D‐glucose in the vicinity of the Cel7A CBM. Panels (a)–(h) display the 2D MDDF for eight distinct α‐D‐glucose concentrations, ranging from 0.1 to 2.0 M. Red regions indicate greater α‐D‐glucose density near the CBM residues.
**Figure S9:** 2D MDDF per residue for α‐D‐mannose in the vicinity of the Cel7A CBM. Panels (a)–(h) display the 2D MDDF for eight distinct α‐D‐mannose concentrations, ranging from 0.1 to 2.0 M. Red regions indicate greater α‐D‐mannose density near the CBM residues.
**Figure S10:** 2D MDDF per residue for α‐D‐xylose in the vicinity of the Cel7A CBM. Panels (a)–(h) display the 2D MDDF for eight distinct α‐D‐xylose concentrations, ranging from 0.1 to 2.0 M. Red regions indicate greater α‐D‐xylose density near the CBM residues.
**Figure S11:** MDDF contributions for the side chains of tyrosine residues (Y5, Y13, Y31, and Y32) in the Cel7A CBM. Rows correspond to residues Y5, Y13, Y31, and Y32 (top to bottom); columns correspond to β‐D‐glucose, β‐D‐mannose, and β‐D‐xylose (left to right). Curves represent sugar concentrations of 0.5 M (green), 1.5 M (red), and 2.0 M (purple).
**Figure S12:** (a)–(c) Occupancy of Y5, Y31, and Y32 in the CBM at 3.0 Å across sugars (glucose, mannose, and xylose) and concentrations (0.5, 1.5, and 2.0). (d)–(f) Normalized occupancy is defined as the occupancy divided by the average bulk density of the sugar in each system.
**Figure S13:** Mean intermittent correlation curves for tyrosines Tyr5, Tyr31, and Tyr32 (top to bottom) as a function of time, at cosolvent concentrations of 0.5, 1.5, and 2.0 M (left to right). Each panel shows the mean curve (solid line) and standard deviation (shaded area) calculated from 20 independent replicas for each cosolvent: β‐D‐glucose (blue), β‐D‐mannose (orange), and β‐D‐xylose (green). Curves were fit to a triexponential decay model (*n* = 3).
**Figure S14:** Average α‐helical content per residue for the Cel7A CD. Data represent the mean across five independent replicas. α‐helical profiles for the CD in water (black), β‐D‐glucose (blue), β‐D‐mannose (orange), and β‐D‐xylose (green) are shown for comparison. Panels (a)–(c) display the results for sugar concentrations of 0.5, 1.0, and 2.0 M, respectively. Gray dotted lines delimit the loop regions.
**Figure S15:** Average β‐sheet content per residue for the Cel7A CD. Data represent the mean across five independent replicas. β‐sheet profiles for the CD in water (black), β‐D‐glucose (blue), β‐D‐mannose (orange), and β‐D‐xylose (green) are shown for comparison. Panels (a)–(c) display the results for sugar concentrations of 0.5, 1.0 and 2.0 M, respectively. Gray dotted lines delimit the loop regions.
**Figure S16:** Average Root Mean Square Fluctuation (RMSF) per residue for the Cel7A CD. Data represent the mean across five independent replicas. WASA profiles for the CD in water (black), β‐D‐glucose (blue), β‐D‐mannose (orange), and β‐D‐xylose (green) are shown for comparison. Panels (a)–(c) display the results for sugar concentrations of 0.5, 1.0, and 2.0 M, respectively. Gray dotted lines delimit the loop regions, while red dotted lines indicate the positions of W38, W40, W367, and W376.
**Figure S17:** Kernel density estimation (KDE) of the B3 loop RMSD. Distributions are shown for pure water (black) and β‐D‐glucose (blue), β‐D‐mannose (orange), and β‐D‐xylose (green) solutions at (a) 0.5 M, (b) 1.0 M, and (c) 2.0 M.
**Figure S18:** Average water accessible surface area (WASA) per residue for the Cel7A CD. Data represent the mean across five independent replicas. WASA profiles for the CD in water (black), β‐D‐glucose (blue), β‐D‐mannose (orange), and β‐D‐xylose (green) are shown for comparison. Panels (a)–(c) display the results for sugar concentrations of 0.5, 1.0, and 2.0 M, respectively. Gray dotted lines delimit the loop regions, while red dotted lines indicate the positions of the W38, W40, W367, and W376.
**Figure S19.1:** Contributions of tunnel residues (within 5 Å of the substrate path) of the Cel7A CD to the β‐D‐glucose MDDF. Panels (a)–(c) display the MDDFs for three distinct β‐D‐glucose concentrations (0.5, 1.0, and 2.0 M). The color scale ranges from 0.0 (white) to 0.025 (red), where red regions indicate greater β‐D‐glucose density near specific CD residues.
**Figure S19.2:** Contributions of tunnel residues (within 5 Å of the substrate path) of the Cel7A CD to the α‐D‐glucose MDDF. The color scale ranges from 0.0 (white) to 0.025 (red), where red regions indicate greater α‐D‐glucose density near specific CD residues.
**Figure S20.1:** Contributions of tunnel residues (within 5 Å of the substrate path) of the Cel7A CD to the β‐D‐mannose MDDF. Panels (a)–(c) display the MDDFs for three distinct β‐D‐mannose concentrations (0.5, 1.0, and 2.0 M). The color scale ranges from 0.0 (white) to 0.025 (red), where red regions indicate greater β‐D‐mannose density near specific CD residues.
**Figure S20.2:** Contributions of tunnel residues (within 5 Å of the substrate path) of the Cel7A CD to the α‐D‐mannose MDDF. The color scale ranges from 0.0 (white) to 0.025 (red), where red regions indicate greater α‐D‐mannose density near specific CD residues.
**Figure S21.1:** Contributions of tunnel residues (within 5 Å of the substrate path) of the Cel7A CD to the β‐D‐xylose MDDF. Panels (a)–(c) display the MDDFs for three distinct β‐D‐xylose concentrations (0.5, 1.0, and 2.0 M). The color scale ranges from 0.0 (white) to 0.025 (red), where red regions indicate greater β‐D‐xylose density near specific CD residues.
**Figure S21.2:** Contributions of tunnel residues (within 5 Å of the substrate path) of the Cel7A CD to the α‐D‐xylose MDDF. The color scale ranges from 0.0 (white) to 0.025 (red), where red regions indicate greater α‐D‐xylose density near specific CD residues.
**Figure S22.1:** Differential β‐D‐glucose vs. β‐D‐mannose MDDF contributions for the Cel7A CD tunnel residues (within 5 Å of the substrate path). Panels (a)–(c) show the difference in residue‐specific contributions at 0.5, 1.0, and 2.0 M. Red regions indicate higher β‐D‐glucose density, whereas blue regions represent a higher β‐D‐mannose density. White regions denote similar occupancy for both sugars.
**Figure S22.2:** Differential α‐D‐glucose vs. α‐D‐mannose MDDF contributions for the Cel7A CD tunnel residues (within 5 Å of the substrate path). Red regions indicate higher α‐D‐glucose density, whereas blue regions represent a higher α‐D‐mannose density. White regions denote similar occupancy for both sugars.
**Figure S23.1:** Differential β‐D‐glucose vs. β‐D‐xylose MDDF contributions for the Cel7A CD tunnel residues (within 5 Å of the substrate path). Panels (a)–(c) show the difference in residue‐specific contributions at 0.5, 1.0, and 2.0 M. Red regions indicate higher β‐D‐glucose density, whereas blue regions represent a higher β‐D‐xylose density. White regions denote similar occupancy for both sugars.
**Figure S23.2:** Differential α‐D‐glucose vs. α‐D‐xylose MDDF contributions for the Cel7A CD tunnel residues (within 5 Å of the substrate path). Red regions indicate higher α‐D‐glucose density, whereas blue regions represent a higher α‐D‐xylose density. White regions denote similar occupancy for both sugars.
**Figure S24:** (a)–(d) Occupancy of W38, W40, W367, and W376 in the CD at 3.0 Å across sugars (β‐D‐glucose, β‐D‐mannose, and β‐D‐xylose) and concentrations (0.5, 1.0, and 2.0). (e)–(h) Normalized occupancy is defined as the occupancy divided by the average bulk density of the sugar in each system.

## Data Availability

Data and scripts necessary to reproduce the results reported in this study are available at a Zenodo repository under DOI: https://doi.org/10.5281/zenodo.19501647.
