# Supplementary material for: Distinct roles of monosaccharides in the inhibition of the carbohydrate‐binding module and catalytic domain of Cel7A
Source: Protein Sci. 2026 Nov 9;35(12):e70848. doi: 10.1002/pro.70848 (PMC13746326; doi:10.1002/pro.70848)
Supplement: Supplementary file 1 [file PRO-35-e70848-s001.pdf]

Supplementary Information

Distinct roles of monosaccharides in the inhibition of the Carbohydrate-Binding Module and Catalytic Domain of Cel7A

Ander F. Pereira, Leandro Martínez, Munir S. Skaf**

Institute of Chemistry and Center for Computing in Engineering & Science, Universidade Estadual de Campinas (UNICAMP), 13083-861 Campinas, SP, Brazil

---

Corresponding authors: lmartine@unicamp.br ; skaf@unicamp.br

Contents:

1. System composition and simulation box parameters for the Cel7A CBM and CD (Tables S1 and S2).
2. Preferential interaction parameters (Γ) of the CBM and CD for β- and α-anomers of the glucose, mannose, and xylose (Tables S3-S4).
3. Ensemble-averaged preferential interaction parameters (Γ) of the CBM and CD for β- and α-anomers of glucose, mannose, and xylose (Table S4).
4. Occupancy and normalized occupancy of Y5, Y31, and Y32 in the CBM across sugars and concentrations (Tables S5-S6 and Figure S12).
5. Structural representation of the sugars studied as cosolvents (Figure S1).
6. 2D MDDF per residue for β-D-glucose, β-D-mannose, and β-D-xylose in the vicinity of the Cel7A CBM (Figures S2, S6, and S7).
7. Solvation structures of the Cel7A CBM in β-D-mannose and β-D-xylose solutions (Figures S3.1 and S4.1).
8. Solvation structures of the Cel7A CBM in α-D-mannose and α-D-xylose solutions (Figure S3.2 and S4.2).

9. Decomposition of the total MDDFs of sugars relative to the CBM into polar, nonpolar, and tyrosine (Tyr) contributions (Figure S5).
10. 2D MDDF per residue for α-D-glucose, α-D-mannose, and α-D-xylose in the vicinity of the Cel7A CBM (Figures S8, S9, and S10).
11. MDDF contributions for the side chains of tyrosine residues (Y5, Y13, Y31, and Y32) in the Cel7A CBM (Figure S11).
12. Mean intermittent correlation curves for tyrosines Y5, Y31, and Y32 as a function of time at distinct sugar concentrations (Figure S13).
13. Average α-helical and β-sheet content per residue for the Cel7A CD (Figures S14 and S15).
14. Average Root Mean Square Fluctuation (RMSF) per residue for the Cel7A CD (Figure S16).
15. Kernel Density Estimation (KDE) of the B3 loop RMSD in pure water and sugar solutions (Figure S17).
16. Average Water Accessible Surface Area (WASA) per residue for the Cel7A CD (Figure S18).
17. Contributions of Cel7A CD tunnel residues to the β-D-glucose, β-D-mannose, and β-D-xylose MDDFs (Figures S19.1, S20.1, and S21.1).
18. Differential β-D-glucose vs. β-D-mannose and β-D-glucose vs. β-D-xylose MDDF contributions for the Cel7A CD tunnel residues (Figures S22.1 and S23.1).
19. Contributions of Cel7A CD tunnel residues to the α-D-glucose, α-D-mannose, and α-D-xylose MDDFs (Figures S19.2, S20.2, and S21.2).
20. Differential α-D-glucose vs. α-D-mannose and α-D-glucose vs. α-D-xylose MDDF contributions for the Cel7A CD tunnel residues (Figures S22.2 and S23.2).
21. Occupancy and normalized occupancy of W38, W40, W367, and W376 in the CD across sugars and concentrations (Figure S24).

Table S1. System composition and simulation box parameters for the Cel7A CBM in the presence of distinct concentrations (0.1-2.0 M) of sugars (glucose, mannose, and xylose). Each system consists of 20 independent production replicates of 80 ns each, totaling 1.6 μs of simulation time per concentration.

Protein domain/ cosolvent	Cosolvent concentration (M)	Number of sugar molecules	Number of water molecules	Box sides (Å)	Simulation time (ns)
CBM / Glucose	0.1	13	7114	60 x 60 x 60	20 x 100
	0.2	25	6762	60 x 60 x 60	20 x 100
	0.3	38	6774	60 x 60 x 60	20 x 100
	0.4	50	6606	60 x 60 x 60	20 x 100
	0.5	63	6580	60 x 60 x 60	20 x 100
	1.0	126	6180	60 x 60 x 60	20 x 100
	1.5	189	5774	60 x 60 x 60	20 x 100
	2.0	253	5384	60 x 60 x 60	20 x 100
CBM / Mannose	0.1	13	7114	60 x 60 x 60	20 x 100
	0.2	25	6763	60 x 60 x 60	20 x 100
	0.3	38	6774	60 x 60 x 60	20 x 100
	0.4	50	6607	60 x 60 x 60	20 x 100
	0.5	63	6581	60 x 60 x 60	20 x 100
	1.0	126	6183	60 x 60 x 60	20 x 100
	1.5	189	5779	60 x 60 x 60	20 x 100
	2.0	253	5391	60 x 60 x 60	20 x 100
CBM / Xylose	0.1	13	7126	60 x 60 x 60	20 x 100
	0.2	25	6785	60 x 60 x 60	20 x 100
	0.3	38	6809	60 x 60 x 60	20 x 100
	0.4	50	6653	60 x 60 x 60	20 x 100
	0.5	63	6639	60 x 60 x 60	20 x 100
	1.0	126	6303	60 x 60 x 60	20 x 100
	1.5	189	5960	60 x 60 x 60	20 x 100
	2.0	253	5638	60 x 60 x 60	20 x 100

Table S2. System composition and simulation box parameters for the Cel7A CD in water and in the presence of distinct concentrations (0.1-2.0 M) of sugars (glucose, mannose, and xylose). Each system consists of 5 independent production replicates of 1,000 ns each, totaling 5.0 μs of simulation time per concentration.

Protein domain/ cosolvent	Cosolvent concentration (M)	Number of sugar molecules	Number of water molecules	Box sides (Å)	Simulation time (ns)
CD / -	-	-	55321	120 x 120 x 120	5 x 1000
CD / Glucose	0.5	496	51779	120 x 120 x 120	5 x 1000
	1.0	994	48730	120 x 120 x 120	5 x 1000
	2.0	1993	42394	120 x 120 x 120	5 x 1000
CD / Mannose	0.5	496	51778	120 x 120 x 120	5 x 1000
	1.0	994	48755	120 x 120 x 120	5 x 1000
	2.0	1993	42448	120 x 120 x 120	5 x 1000
CD / Xylose	0.5	496	52244	120 x 120 x 120	5 x 1000
	1.0	993	49648	120 x 120 x 120	5 x 1000
	2.0	1991	44344	120 x 120 x 120	5 x 1000

Table S3. Preferential interaction parameters (Γ) of the CBM and CD for β-D-glucose ($\Gamma_{\beta\text{-D-glucose}}$), α-D-glucose ($\Gamma_{\alpha\text{-D-glucose}}$), β-D-mannose ($\Gamma_{\beta\text{-D-mannose}}$), α-D-mannose ($\Gamma_{\alpha\text{-D-mannose}}$), β-D-xylose ($\Gamma_{\beta\text{-D-xylose}}$), and α-D-xylose ($\Gamma_{\alpha\text{-D-xylose}}$) solutions.

**Cosolvent concentration (mol L^{-1})**	**CBM**					
	$\Gamma_{\beta\text{-D-glucose}}$	$\Gamma_{\alpha\text{-D-glucose}}$	$\Gamma_{\beta\text{-D-mannose}}$	$\Gamma_{\alpha\text{-D-mannose}}$	$\Gamma_{\beta\text{-D-xylose}}$	$\Gamma_{\alpha\text{-D-xylose}}$
0.1	0.08 ± 0.03	0.18 ± 0.03	0.12 ± 0.03	0.20 ± 0.03	0.13 ± 0.02	0.24 ± 0.03
0.2	0.16 ± 0.04	0.27 ± 0.04	0.12 ± 0.03	0.40 ± 0.04	0.31 ± 0.04	0.31 ± 0.03
0.3	0.28 ± 0.04	0.33 ± 0.05	0.20 ± 0.03	0.59 ± 0.06	0.30 ± 0.05	0.53 ± 0.04
0.4	0.27 ± 0.05	0.54 ± 0.04	0.31 ± 0.05	0.62 ± 0.04	0.49 ± 0.05	0.78 ± 0.06
0.5	0.31 ± 0.04	0.52 ± 0.06	0.32 ± 0.05	0.71 ± 0.05	0.58 ± 0.05	0.85 ± 0.06
1.0	0.24 ± 0.10	0.79 ± 0.10	0.42 ± 0.1	1.13 ± 0.07	0.84 ± 0.09	1.36 ± 0.08
1.5	0.20 ± 0.10	0.67 ± 0.09	0.21 ± 0.1	1.29 ± 0.10	0.96 ± 0.10	1.46 ± 0.07
2.0	-0.17 ± 0.10	0.51 ± 0.13	-0.20 ± 0.09	1.03 ± 0.10	0.77 ± 0.09	1.36 ± 0.11
	**CD**					
0.5	-4.68 ± 0.19	-	-4.60 ± 0.18	-	-2.91 ± 0.25	-
1.0	-10.46 ± 0.59	-	-10.37 ± 0.49	-	-7.13 ± 0.55	-
2.0	-27.63 ± 0.80	-24.81 ± 0.59	-25.61 ± 0.57	-21.57 ± 0.71	-19.22 ± 0.53	-15.90 ± 0.42

Table S4. Ensemble-averaged preferential interaction parameters (Γ) of the CBM in aqueous solutions of glucose ($\Gamma_{Glucose}$), mannose ($\Gamma_{Mannose}$), and xylose (Γ_{Xylose}). Values represent weighted averages based on experimental equilibrium ratios of β- and α-anomers in water: 64% : 36% for glucose, 35% : 65%% for mannose, and 65% : 35% for xylose. Errors denote standard errors.

Cosolvent concentration (mol L^{-1})	CBM		
	$\Gamma_{Glucose}$	$\Gamma_{Mannose}$	Γ_{Xylose}
0.1	0.12 ± 0.02	0.18 ± 0.02	0.17 ± 0.02
0.2	0.20 ± 0.03	0.30 ± 0.03	0.31 ± 0.03
0.3	0.30 ± 0.03	0.45 ± 0.04	0.38 ± 0.04
0.4	0.37 ± 0.03	0.51 ± 0.03	0.59 ± 0.04
0.5	0.39 ± 0.03	0.57 ± 0.04	0.67 ± 0.04
1.0	0.44 ± 0.08	0.89 ± 0.06	1.03 ± 0.07
1.5	0.36 ± 0.08	0.91 ± 0.07	1.13 ± 0.08
2.0	0.10 ± 0.09	0.60 ± 0.07	0.98 ± 0.08
2.0*	-0.14 ± 0.11	0.53 ± 0.16	0.96 ± 0.13
	CD		
2.0	-26.62 ± 0.55	-22.98 ± 0.5	-18.06 ± 0.38

* Data obtained directly from simulations performed with explicit mixtures of β- and α-anomers.

Table S5. Occupancy of Y5, Y31, and Y32 around CBM at 3.0 Å across sugars (glucose, mannose, and xylose) and concentrations (0.5, 1.5, and 2.0).

Protein domain/ cosolvent	Cosolvent concentration (M)	Occupancy at 3.0 Å		
		Y5	Y31	Y32
CBM / α-D-glucose	0.5	0.44 ± 0.05	0.48 ± 0.05	0.35 ± 0.05
	1.5	1.22 ± 0.07	1.32 ± 0.09	0.99 ± 0.08
	2.0	1.59 ± 0.08	1.75 ± 0.10	1.30 ± 0.11
CBM / α-D-mannose	0.5	0.43 ± 0.04	0.50 ± 0.06	0.38 ± 0.06
	1.5	1.28 ± 0.08	1.41 ± 0.09	1.06 ± 0.10
	2.0	1.64 ± 0.11	1.81 ± 0.14	1.34 ± 0.11
CBM / α-D-xylose	0.5	0.42 ± 0.03	0.48 ± 0.04	0.36 ± 0.05
	1.5	1.16 ± 0.08	1.32 ± 0.10	0.95 ± 0.10
	2.0	1.50 ± 0.08	1.64 ± 0.09	1.17 ± 0.08
CBM / β-D-glucose	0.5	0.40 ± 0.04	0.42 ± 0.05	0.33 ± 0.04
	1.5	1.14 ± 0.08	1.20 ± 0.06	0.89 ± 0.09
	2.0	1.48 ± 0.08	1.63 ± 0.11	1.21 ± 0.14
CBM / β-D-mannose	0.5	0.38 ± 0.03	0.41 ± 0.05	0.31 ± 0.06
	1.5	1.12 ± 0.06	1.22 ± 0.11	0.93 ± 0.12
	2.0	1.46 ± 0.10	1.61 ± 0.20	1.19 ± 0.18
CBM / β-D-xylose	0.5	0.39 ± 0.05	0.40 ± 0.04	0.32 ± 0.05
	1.5	1.09 ± 0.09	1.18 ± 0.08	0.91 ± 0.10
	2.0	1.41 ± 0.08	1.53 ± 0.11	1.09 ± 0.09

Table S6. Normalized occupancy of Y5, Y31, and Y32 in the CBM at 3.5 Å across sugars (glucose, mannose, and xylose) and concentrations (0.5, 1.5, and 2.0). Normalized occupancy is defined as the occupancy divided by the average bulk density of the sugar in each system.

**Protein domain/ cosolvent**	**Cosolvent concentration (M)**	**Normalized Coordination Number at 3.5 Å**		
		**Y5**	**Y31**	**Y32**
CBM / α-D-glucose	0.5	0.87 ± 0.09	0.95 ± 0.10	0.70 ± 0.09
	1.5	0.81 ± 0.05	0.88 ± 0.06	0.66 ± 0.05
	2.0	0.79 ± 0.04	0.87 ± 0.05	0.65 ± 0.06
CBM / α-D-mannose	0.5	0.85 ± 0.08	1.0 ± 0.13	0.76 ± 0.12
	1.5	0.85 ± 0.05	0.94 ± 0.06	0.71 ± 0.06
	2.0	0.82 ± 0.05	0.91 ± 0.07	0.68 ± 0.06
CBM / α-D-xylose	0.5	0.83 ± 0.06	0.95 ± 0.08	0.71 ± 0.09
	1.5	0.77 ± 0.05	0.88 ± 0.06	0.63 ± 0.07
	2.0	0.75 ± 0.04	0.82 ± 0.05	0.59 ± 0.04
CBM / β-D-glucose	0.5	0.79 ± 0.09	0.82 ± 0.09	0.65 ± 0.08
	1.5	0.75 ± 0.05	0.79 ± 0.04	0.59 ± 0.06
	2.0	0.73 ± 0.04	0.81 ± 0.05	0.60 ± 0.07
CBM / β-D-mannose	0.5	0.76 ± 0.07	0.81 ± 0.10	0.62 ± 0.12
	1.5	0.74 ± 0.04	0.80 ± 0.07	0.61 ± 0.08
	2.0	0.72 ± 0.05	0.80 ± 0.10	0.59 ± 0.09
CBM / β-D-xylose	0.5	0.77 ± 0.09	0.79 ± 0.07	0.63 ± 0.09
	1.5	0.72 ± 0.06	0.79 ± 0.05	0.60 ± 0.07
	2.0	0.70 ± 0.04	0.76 ± 0.05	0.55 ± 0.05

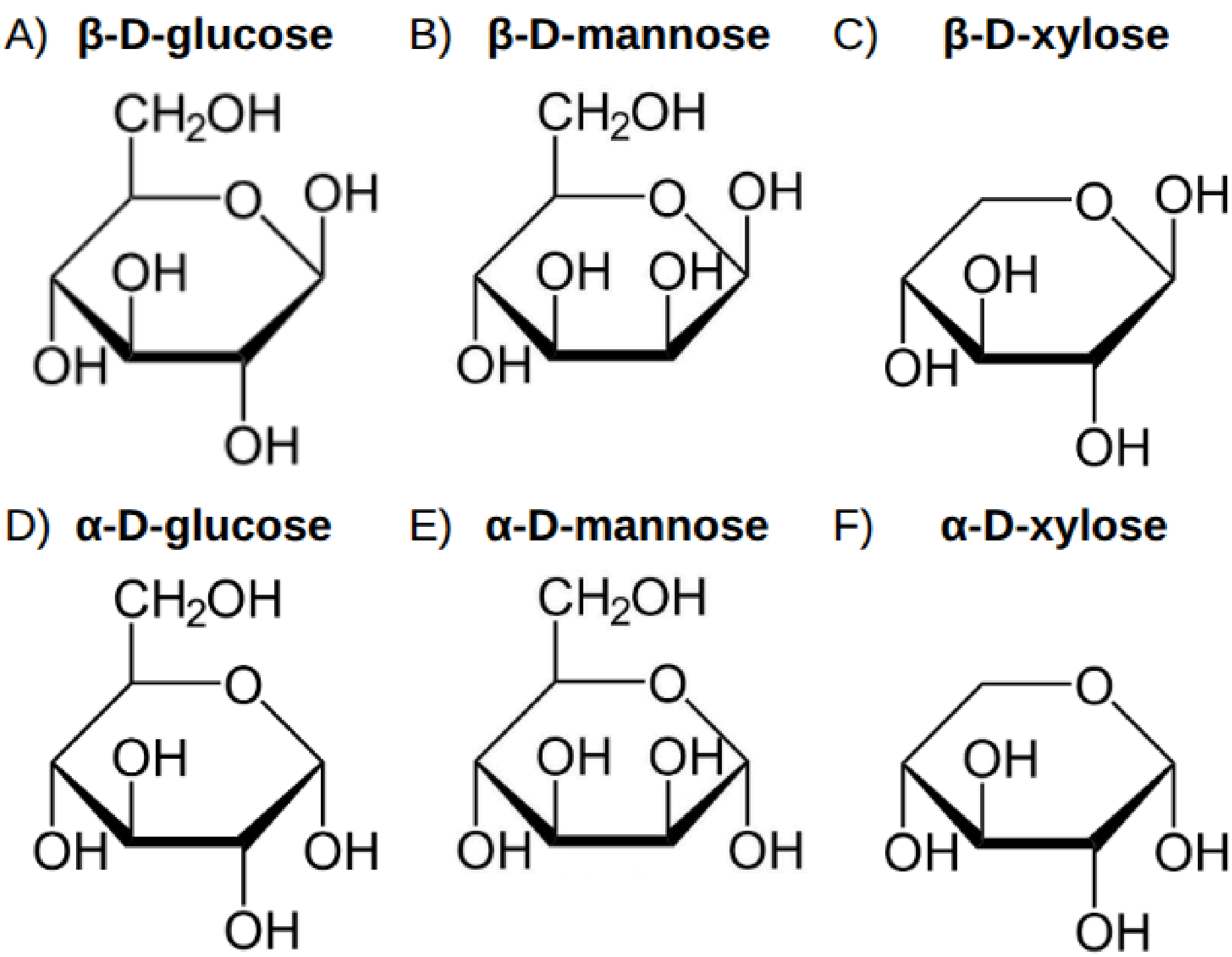

Figure S1. Structural representation of the sugars studied as cosolvents. Panels A)-C) show the chemical structures of A) β-D-glucose, B) β-D-mannose, and C) β-D-xylose, whereas panels D-F show those of D) α-D-glucose, E) α-D-mannose, and F) α-D-xylose.

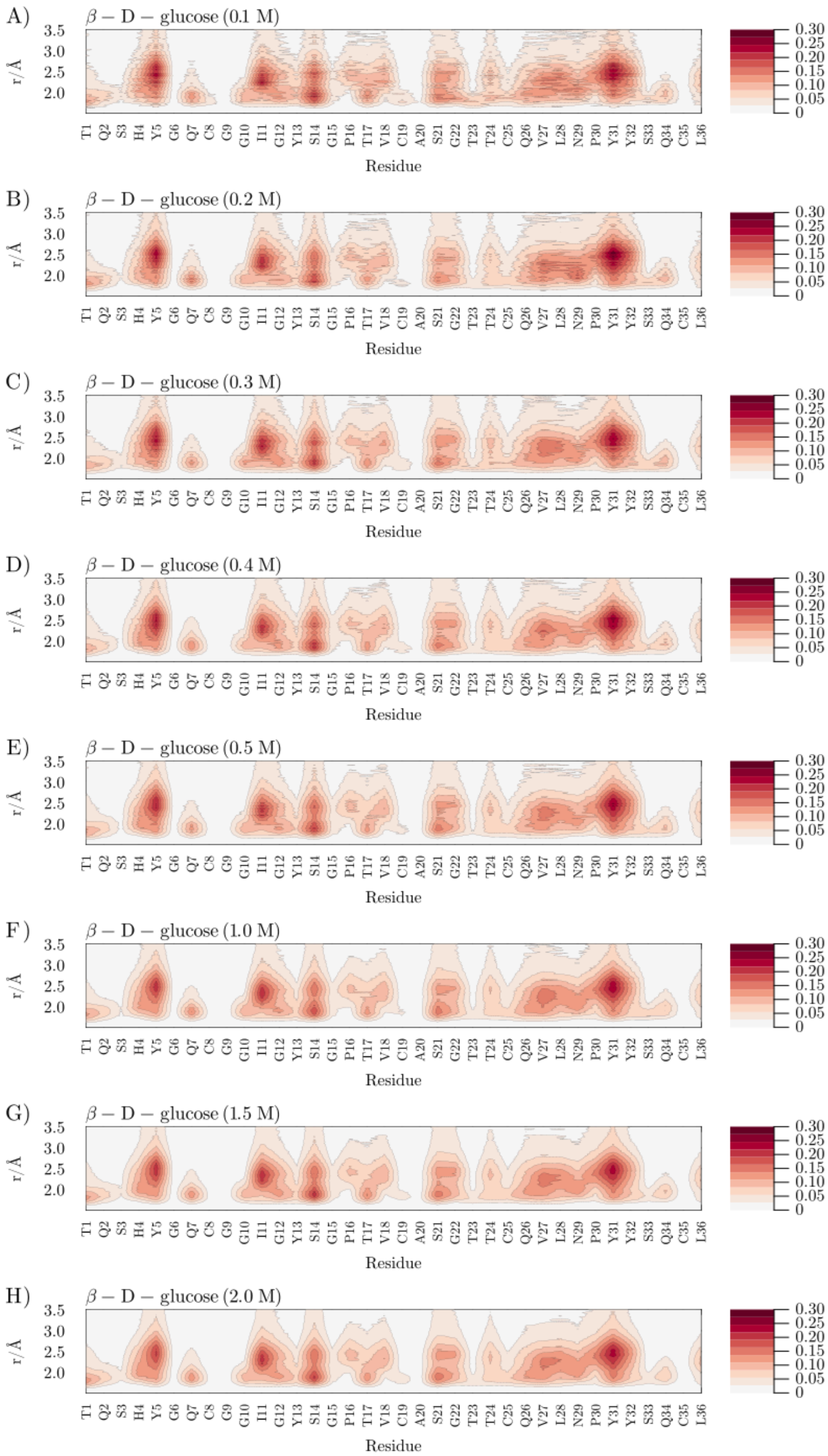

Figure S2. 2D MDDF per residue for β-D-glucose in the vicinity of the Cel7A CBM. Panels A)-H) display the 2D MDDF for eight distinct β-D-glucose concentrations, ranging from 0.1 M to 2.0 M. Red regions indicate greater β-D-glucose density near the CBM residues.

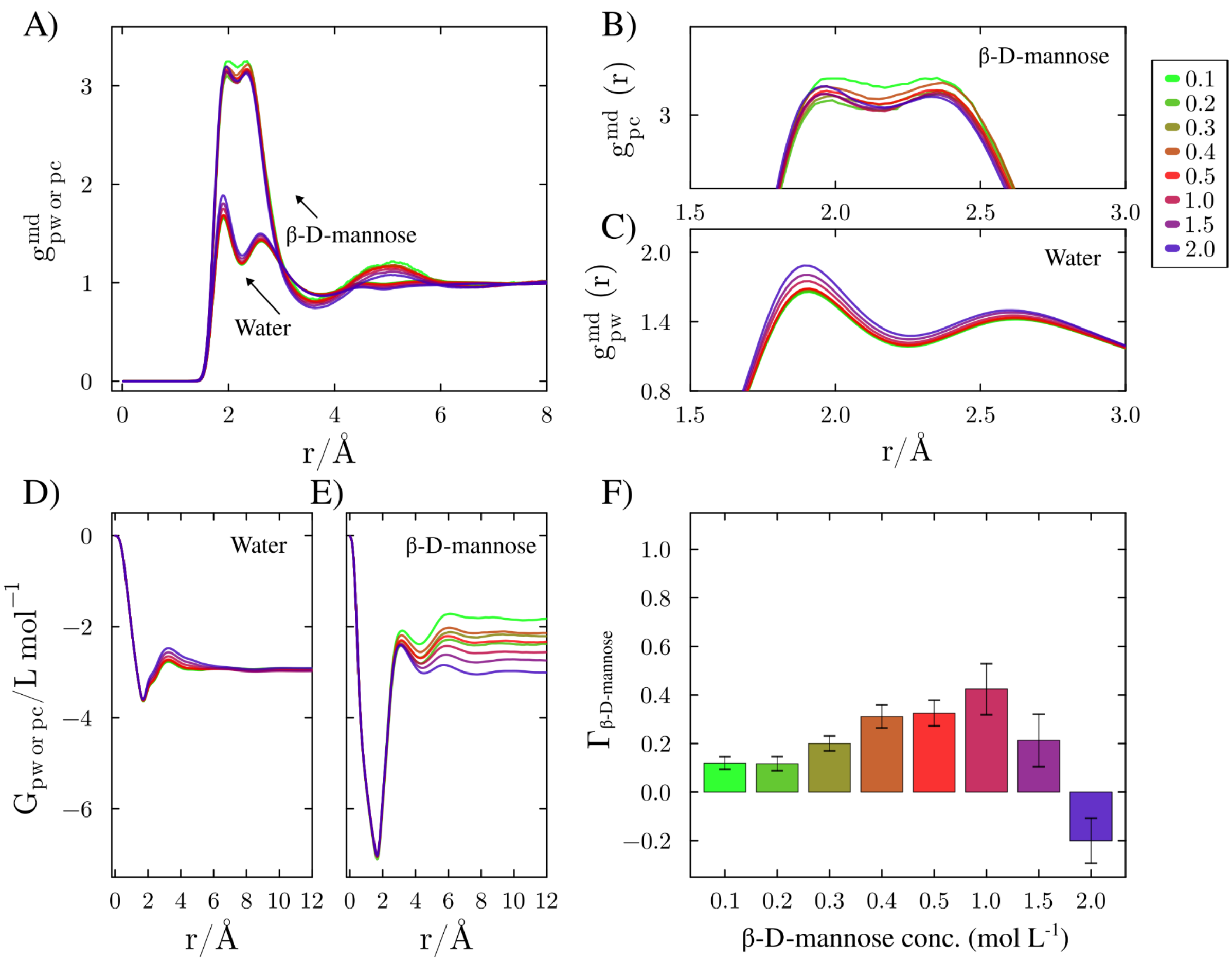

Figure S3.1. Solvation structures of the Cel7A CBM in β-D-mannose solutions. A) MDDFs of water and β-D-mannose for the CBM at eight concentrations ranging from 0.1 M (green) to 2.0 M (purple). B) and C) are the most prominent peaks in the MDDFs of β-D-mannose and water. D) and E) show the KBIs for water and β-D-mannose. F) Preferential interaction parameters (Γ).

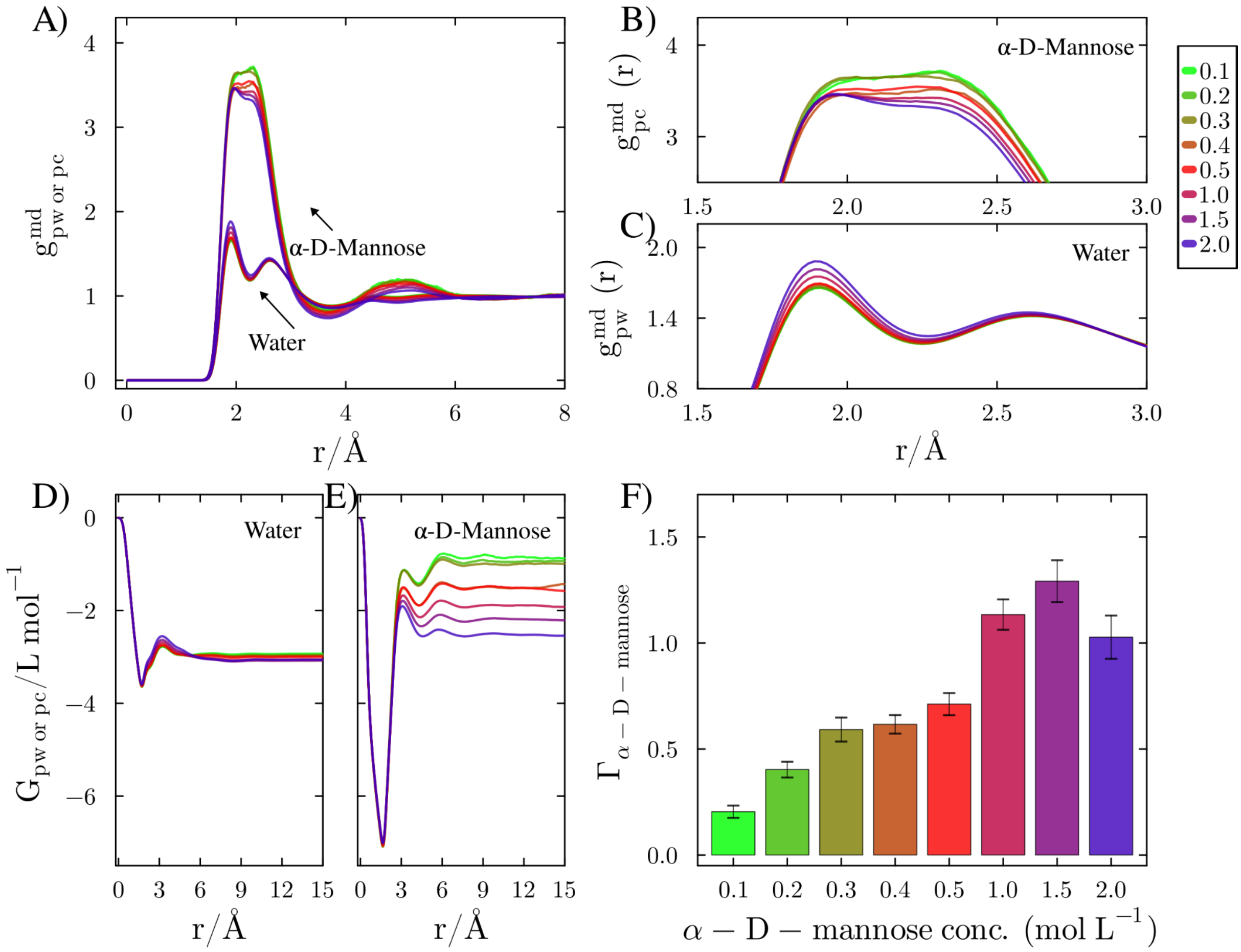

Figure S3.2. Solvation structures of the Cel7A CBM in α-D-mannose solutions. A) MDDFs of water and α-D-mannose for the CBM at eight concentrations ranging from 0.1 M (green) to 2.0 M (purple). B) and C) are the most prominent peaks in the MDDFs of α-D-mannose and water. D) and E) show the KBIs for water and α-D-mannose. F) Preferential interaction parameters (Γ).

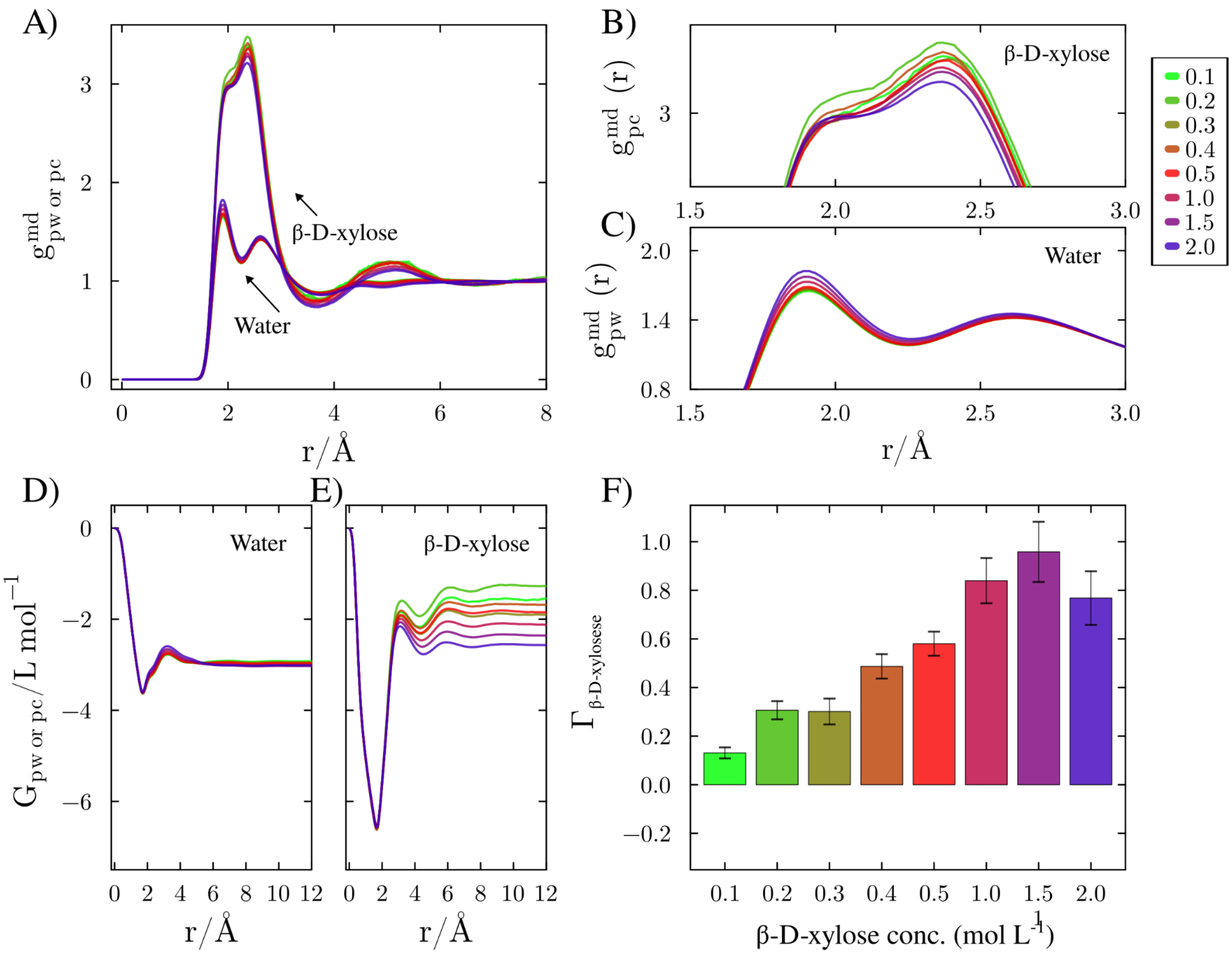

Figure S4.1. Solvation structures of the Cel7A CBM in β-D-xylose solutions. A) MDDFs of water and β-D-xylose for the CBM at eight concentrations ranging from 0.1 M (green) to 2.0 M (purple). B) and C) are the most prominent peaks in the MDDFs of β-D-xylose and water. D) and E) show the KBIs for water and β-D-xylose. F) Preferential interaction parameters (Γ).

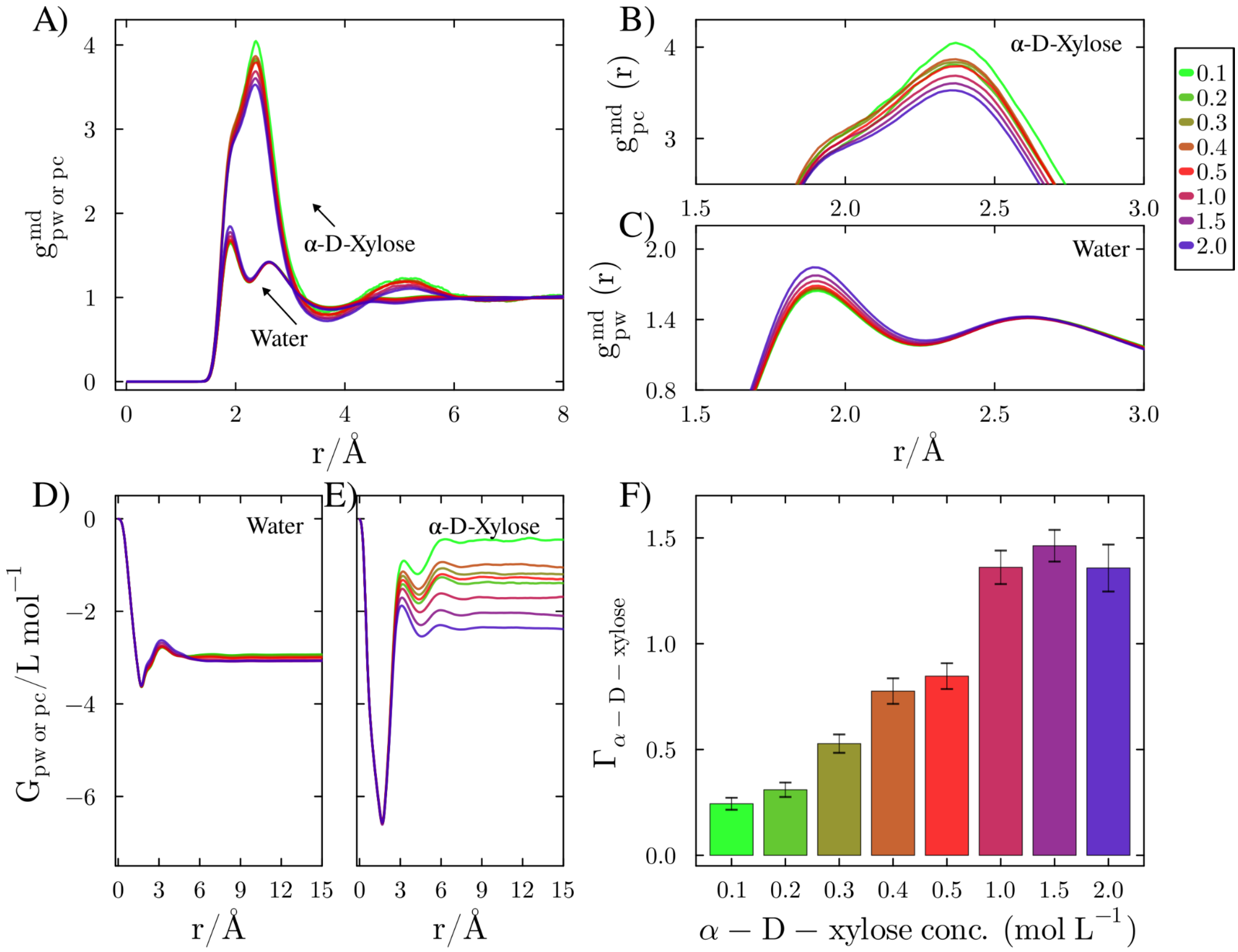

Figure S4.2. Solvation structures of the Cel7A CBM in α-D-xylose solutions. A) MDDFs of water and α-D-xylose for the CBM at eight concentrations ranging from 0.1 M (green) to 2.0 M (purple). B) and C) are the most prominent peaks in the MDDFs of α-D-xylose and water. D) and E) show the KBIs for water and α-D-xylose. F) Preferential interaction parameters (Γ).

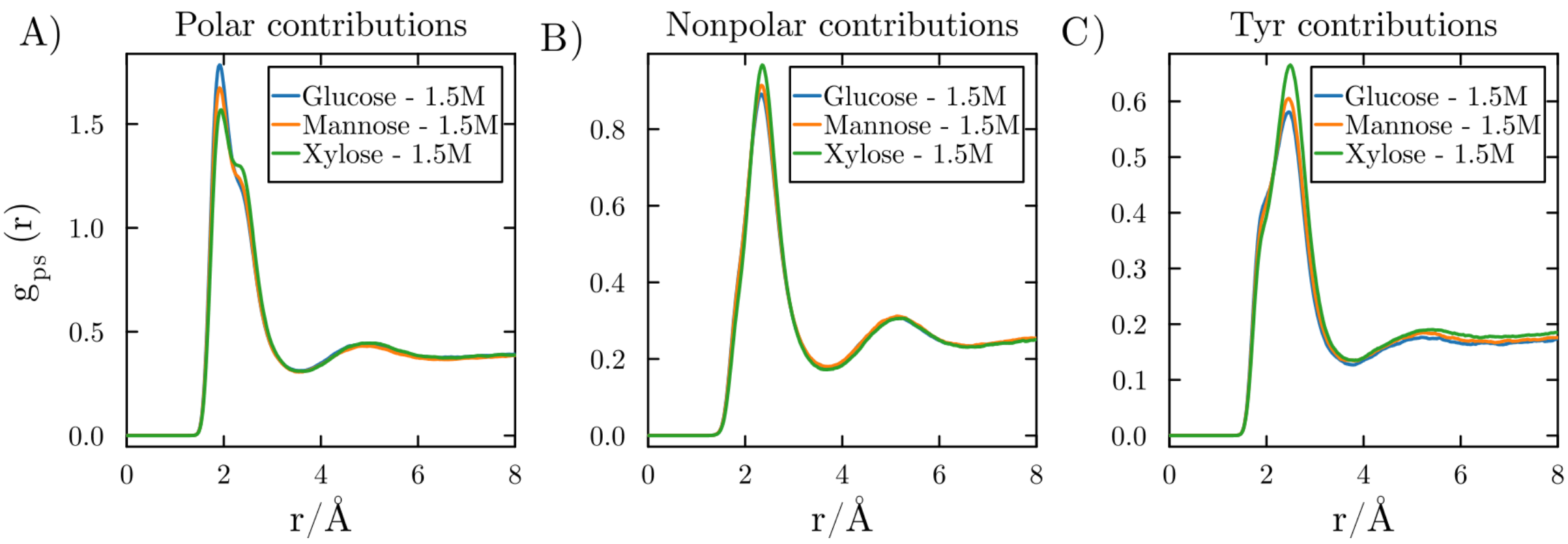

Figure S5. Decomposition of the total MDDFs of A) β-D-glucose (blue), B) β-D-mannose (orange), and C) β-D-xylose (green) at 1.5 M relative to the CBM, showing polar, nonpolar, and tyrosine (Tyr) contributions.

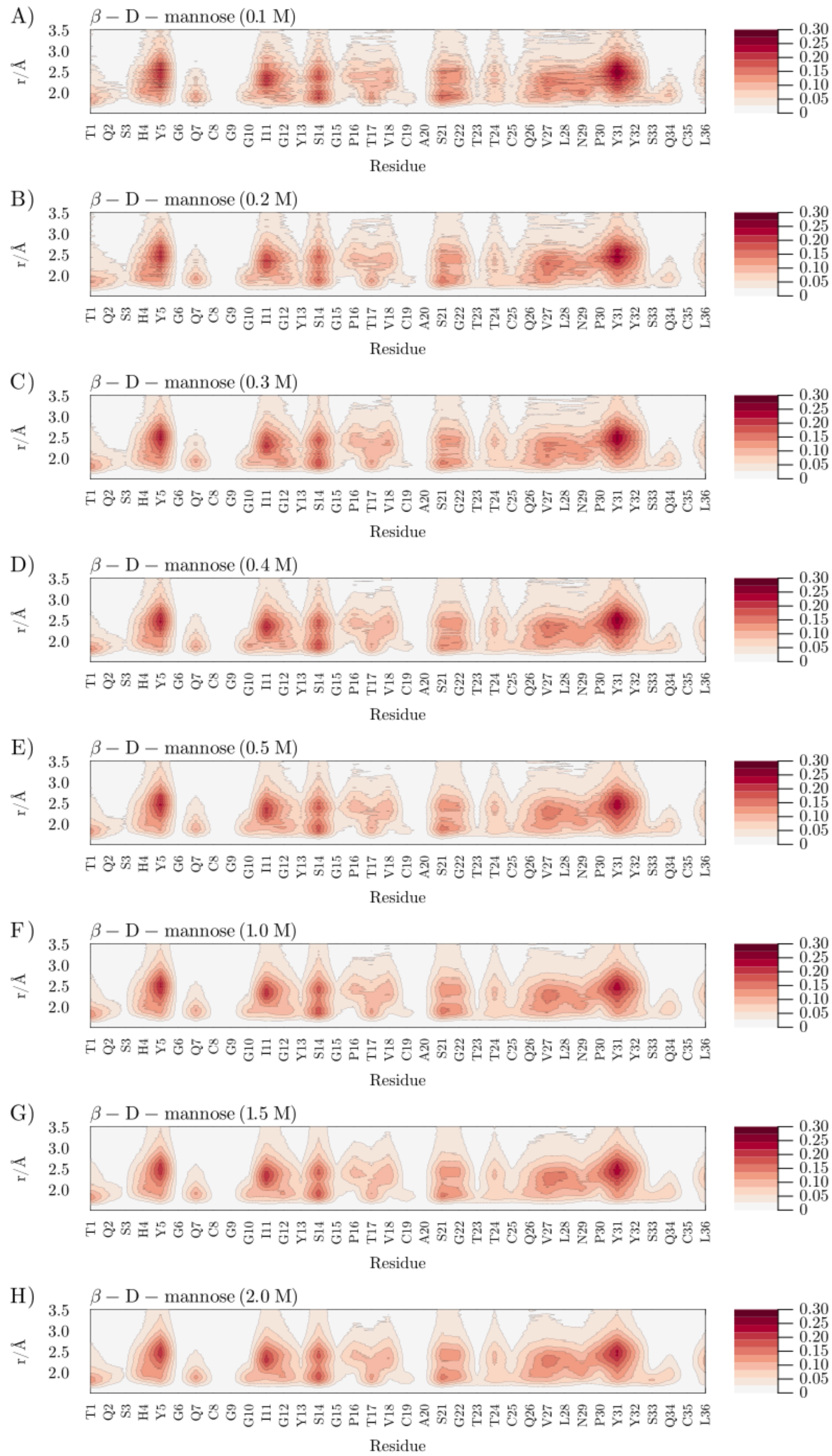

Figure S6. 2D MDDF per residue for β-D-mannose in the vicinity of the Cel7A CBM. Panels A)-H) display the 2D MDDF for eight distinct β-D-mannose concentrations, ranging from 0.1 M to 2.0 M. Red regions indicate greater β-D-mannose density near the CBM residues.

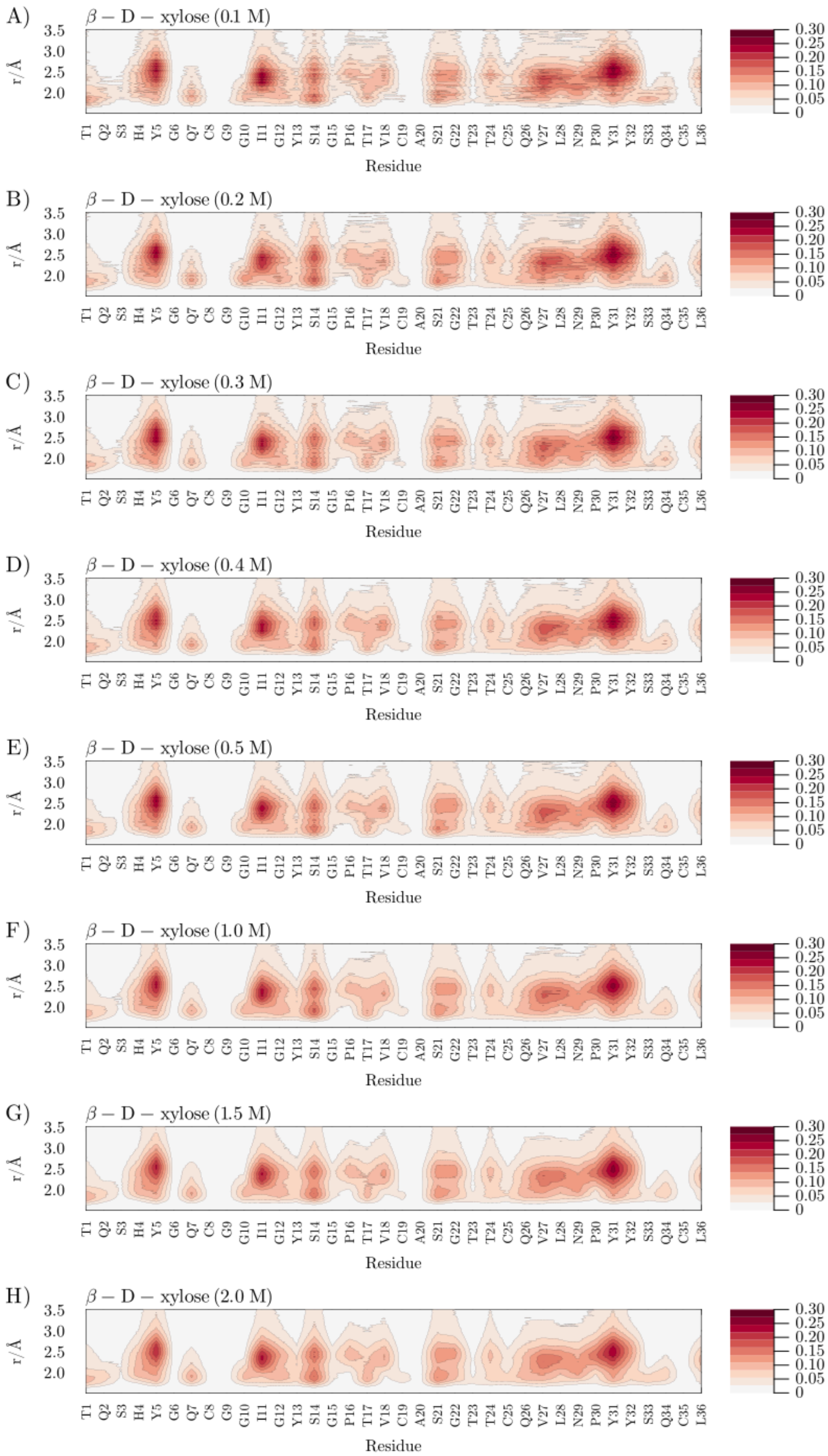

Figure S7. 2D MDDF per residue for β-D-xylose in the vicinity of the Cel7A CBM. Panels A)-H) display the 2D MDDF for eight distinct β-D-xylose concentrations, ranging from 0.1 M to 2.0 M. Red regions indicate greater β-D-xylose density near the CBM residues.

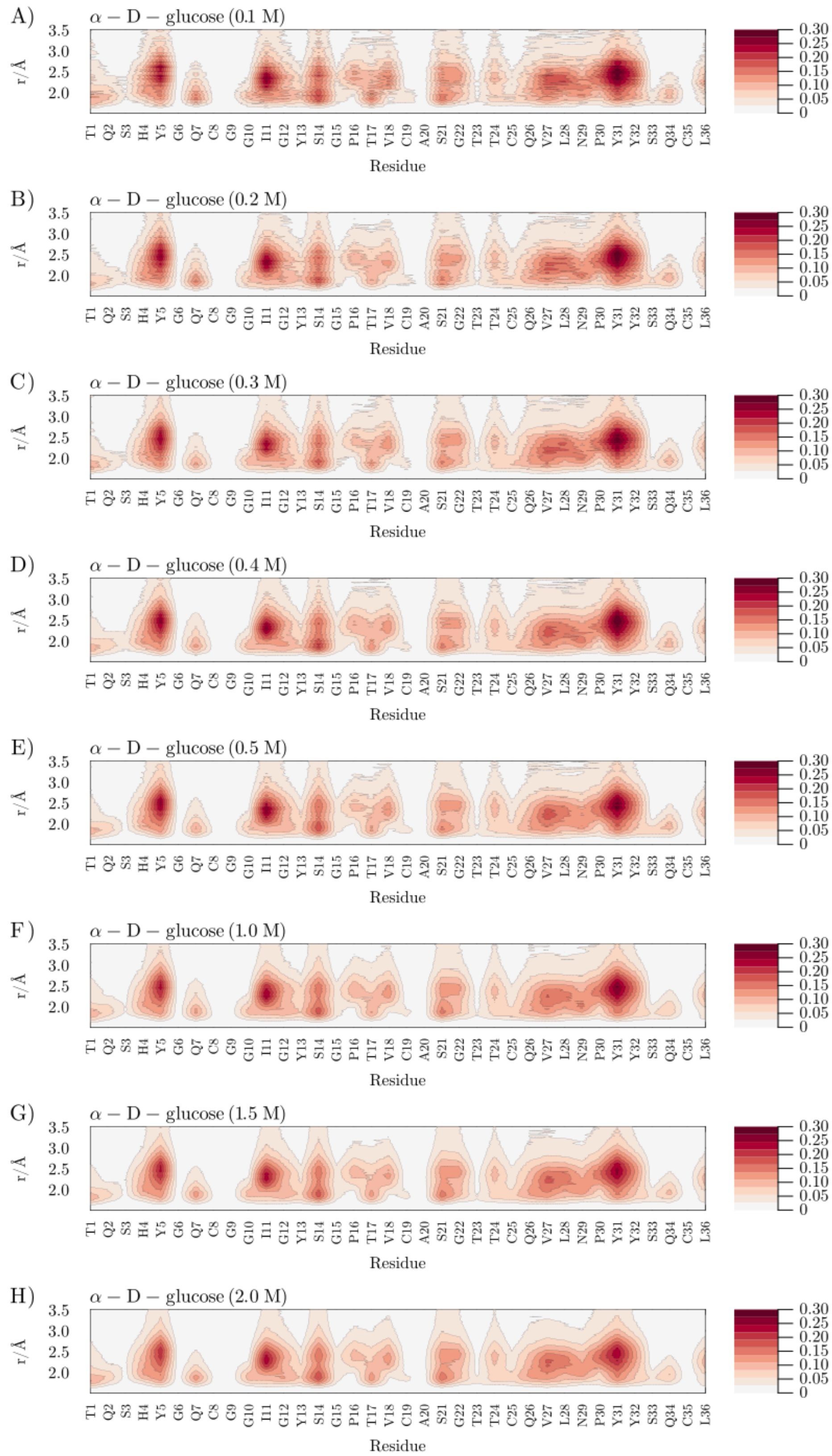

Figure S8. 2D MDDF per residue for α-D-glucose in the vicinity of the Cel7A CBM. Panels A)-H) display the 2D MDDF for eight distinct α-D-glucose concentrations, ranging from 0.1 M to 2.0 M. Red regions indicate greater α-D-glucose density near the CBM residues.

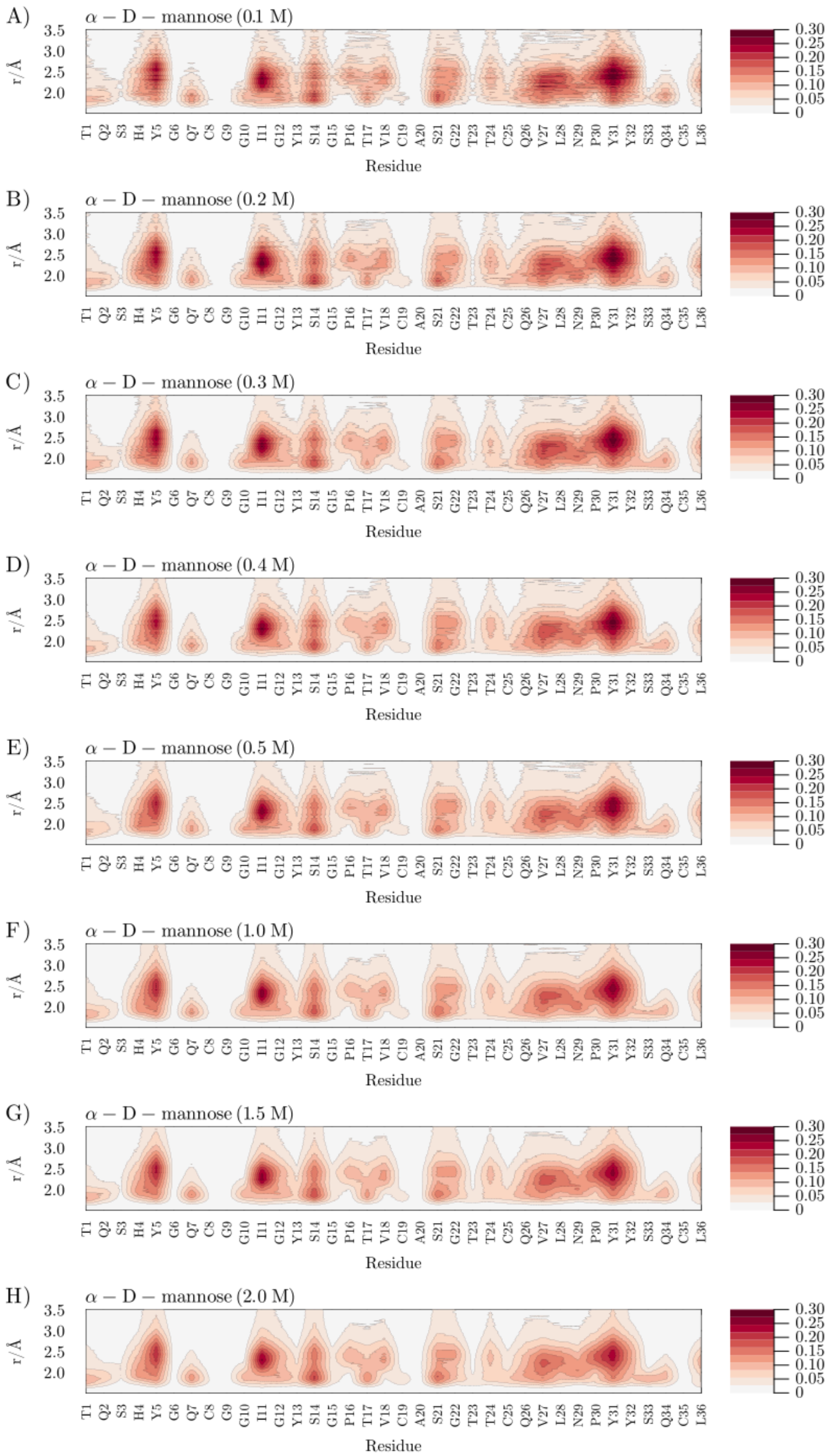

Figure S9. 2D MDDF per residue for α-D-mannose in the vicinity of the Cel7A CBM. Panels A)-H) display the 2D MDDF for eight distinct α-D-mannose concentrations, ranging from 0.1 M to 2.0 M. Red regions indicate greater α-D-mannose density near the CBM residues.

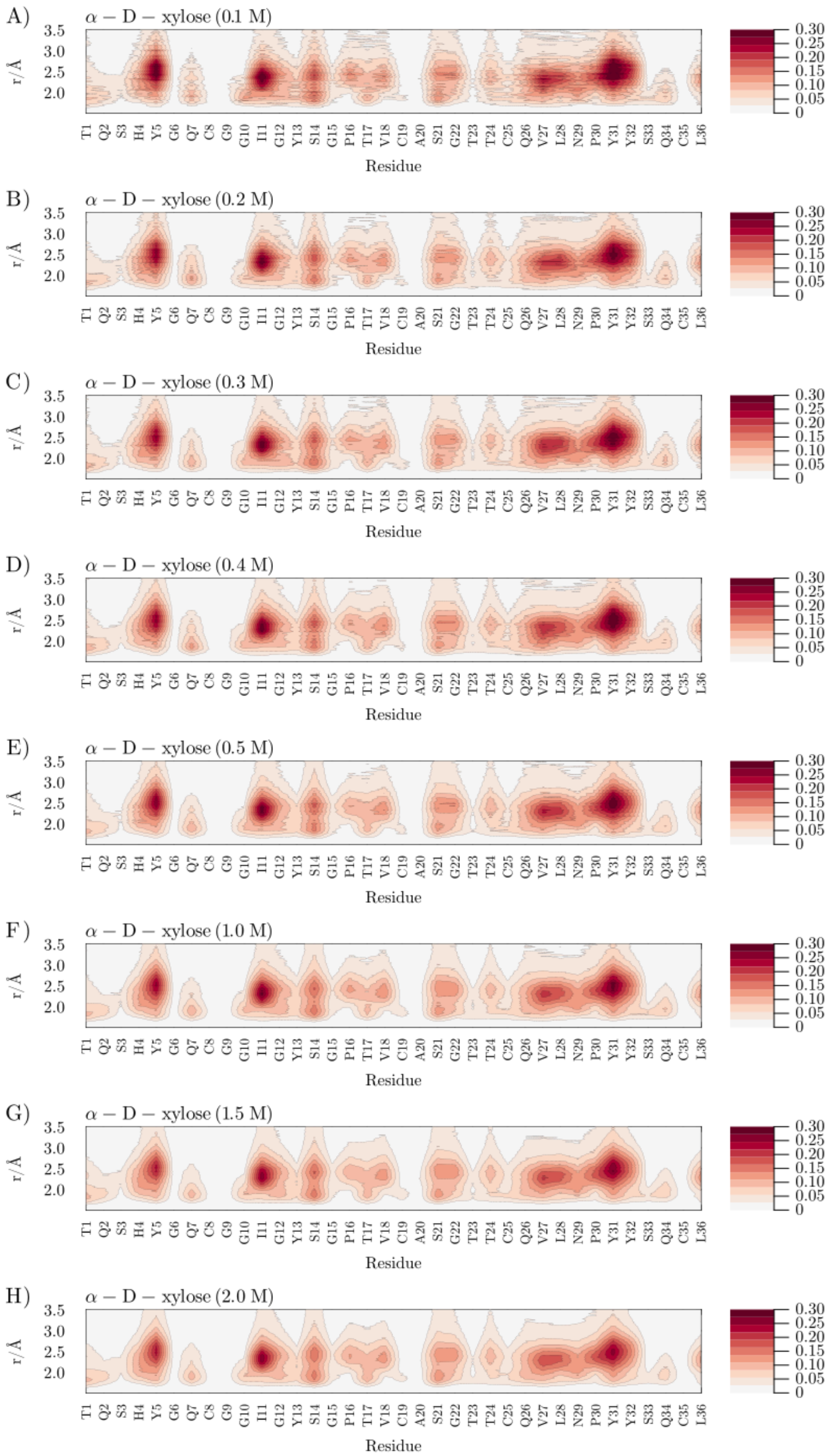

Figure S10. 2D MDDF per residue for α-D-xylose in the vicinity of the Cel7A CBM. Panels A)-H) display the 2D MDDF for eight distinct α-D-xylose concentrations, ranging from 0.1 M to 2.0 M. Red regions indicate greater α-D-xylose density near the CBM residues.

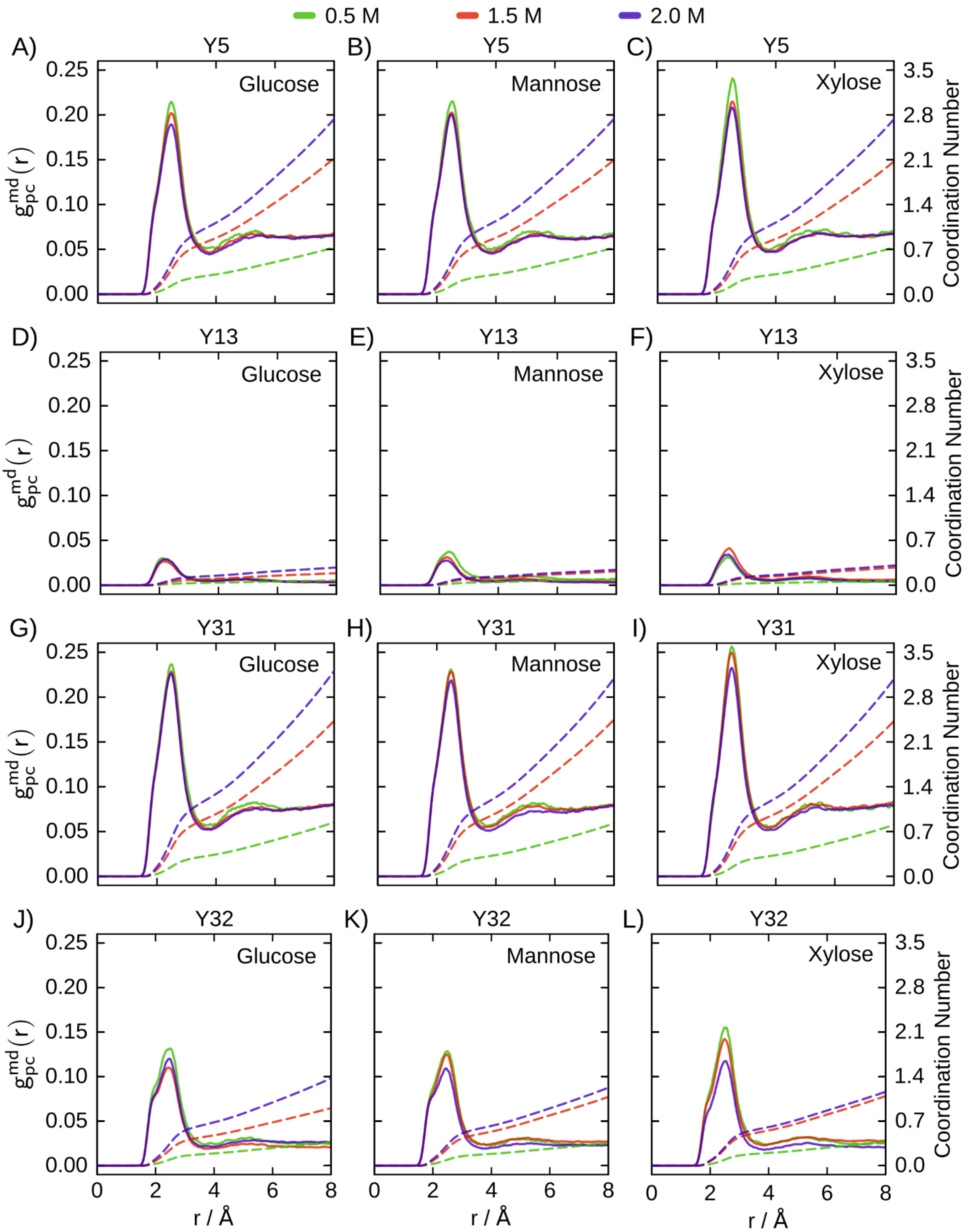

Figure S11. MDDF contributions for the side chains of tyrosine residues (Y5, Y13, Y31, and Y32) in the Cel7A CBM. Rows correspond to residues Y5, Y13, Y31, and Y32 (top to bottom); columns correspond to β-D-glucose, β-D-mannose, and β-D-xylose (left to right). Curves represent sugar concentrations of 0.5 M (green), 1.5 M (red), and 2.0 M (purple).

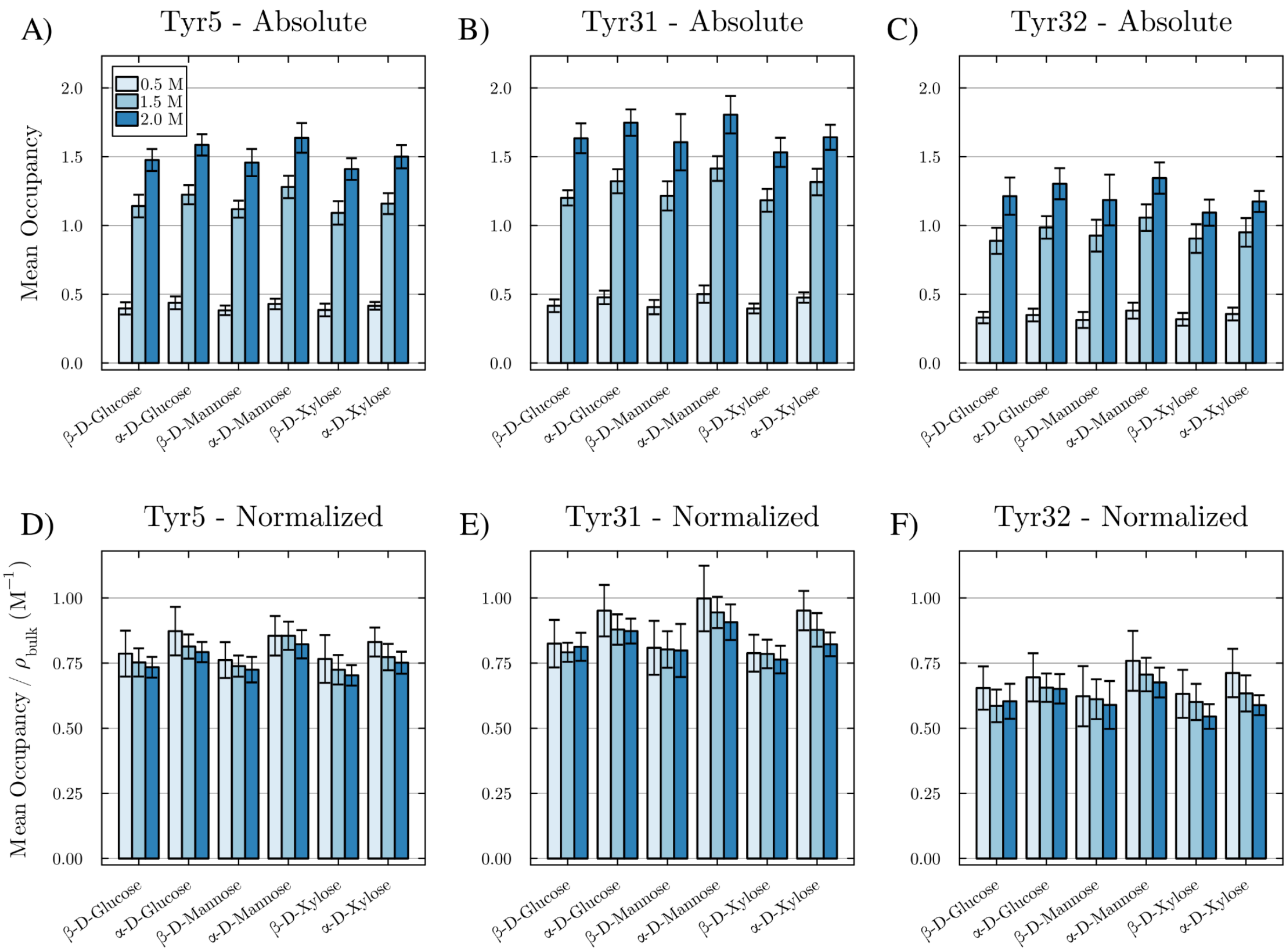

Figure S12. A)-C) Occupancy of Y5, Y31, and Y32 in the CBM at 3.0 Å across sugars (glucose, mannose, and xylose) and concentrations (0.5, 1.5, and 2.0). D)-F) Normalized occupancy is defined as the occupancy divided by the average bulk density of the sugar in each system.

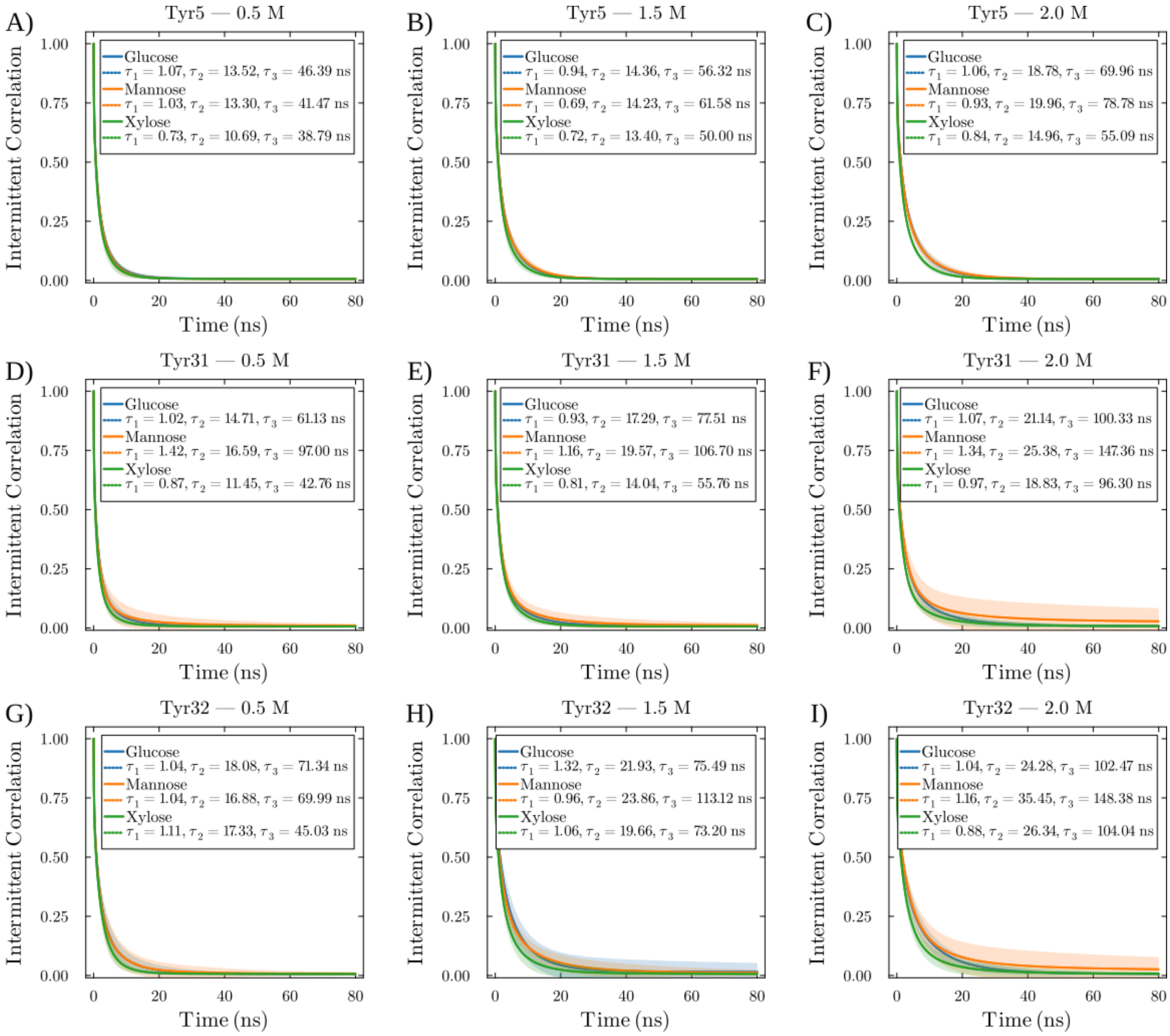

Figure S13. Mean intermittent correlation curves for tyrosines Tyr5, Tyr31, and Tyr32 (top to bottom) as a function of time, at cosolvent concentrations of 0.5, 1.5, and 2.0 M (left to right). Each panel shows the mean curve (solid line) and standard deviation (shaded area) calculated from 20 independent replicas for each cosolvent: β-D-glucose (blue), β-D-mannose (orange), and β-D-xylose (green). Curves were fit to a triexponential decay model (n = 3).

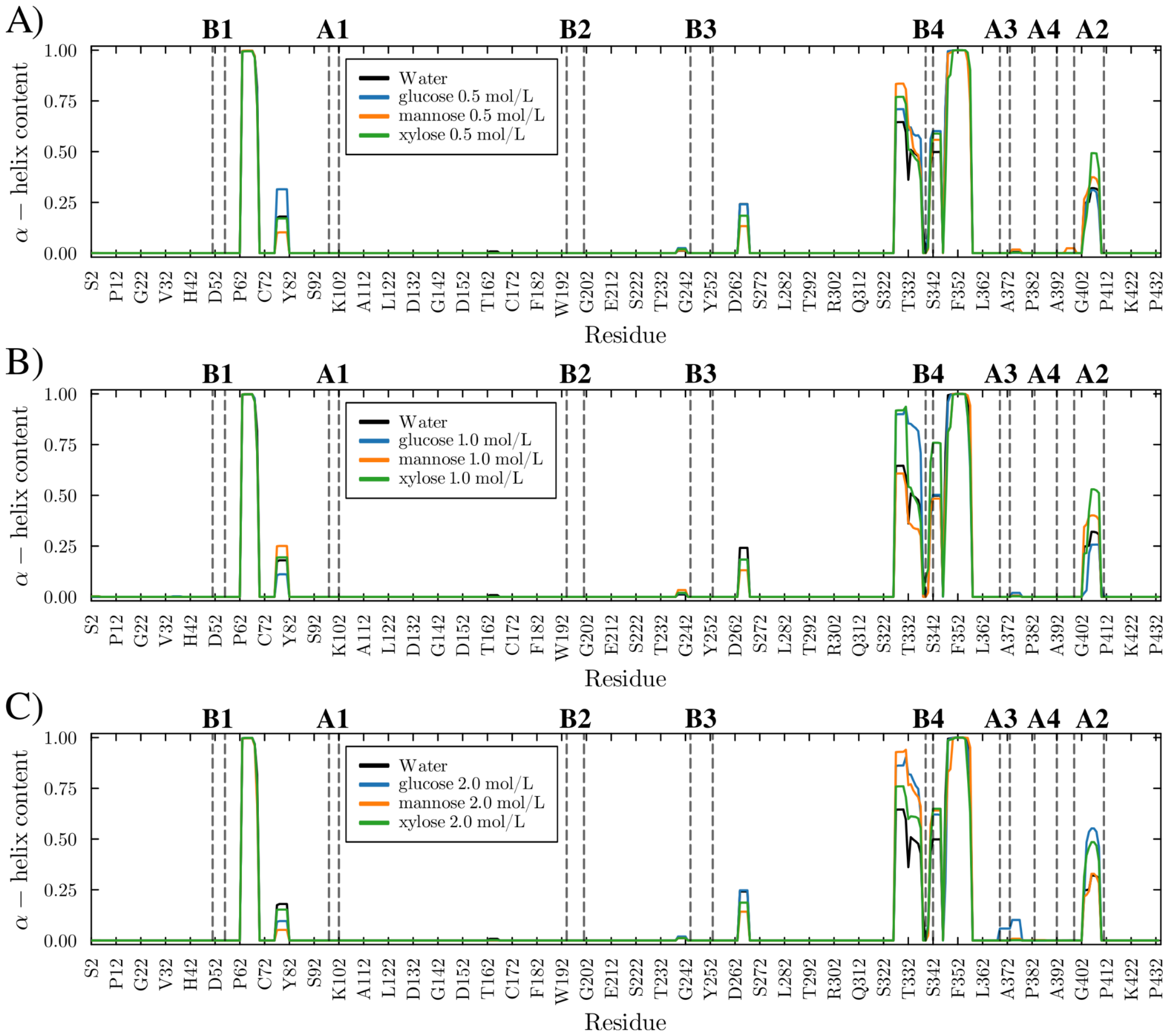

Figure S14. Average α-helical content per residue for the Cel7A CD. Data represent the mean across five independent replicas. α-helical profiles for the CD in water (black), β-D-glucose (blue), β-D-mannose (orange), and β-D-xylose (green) are shown for comparison. Panels A)-C) display the results for sugar concentrations of 0.5 M, 1.0 M, and 2.0 M, respectively. Gray dotted lines delimit the loop regions.

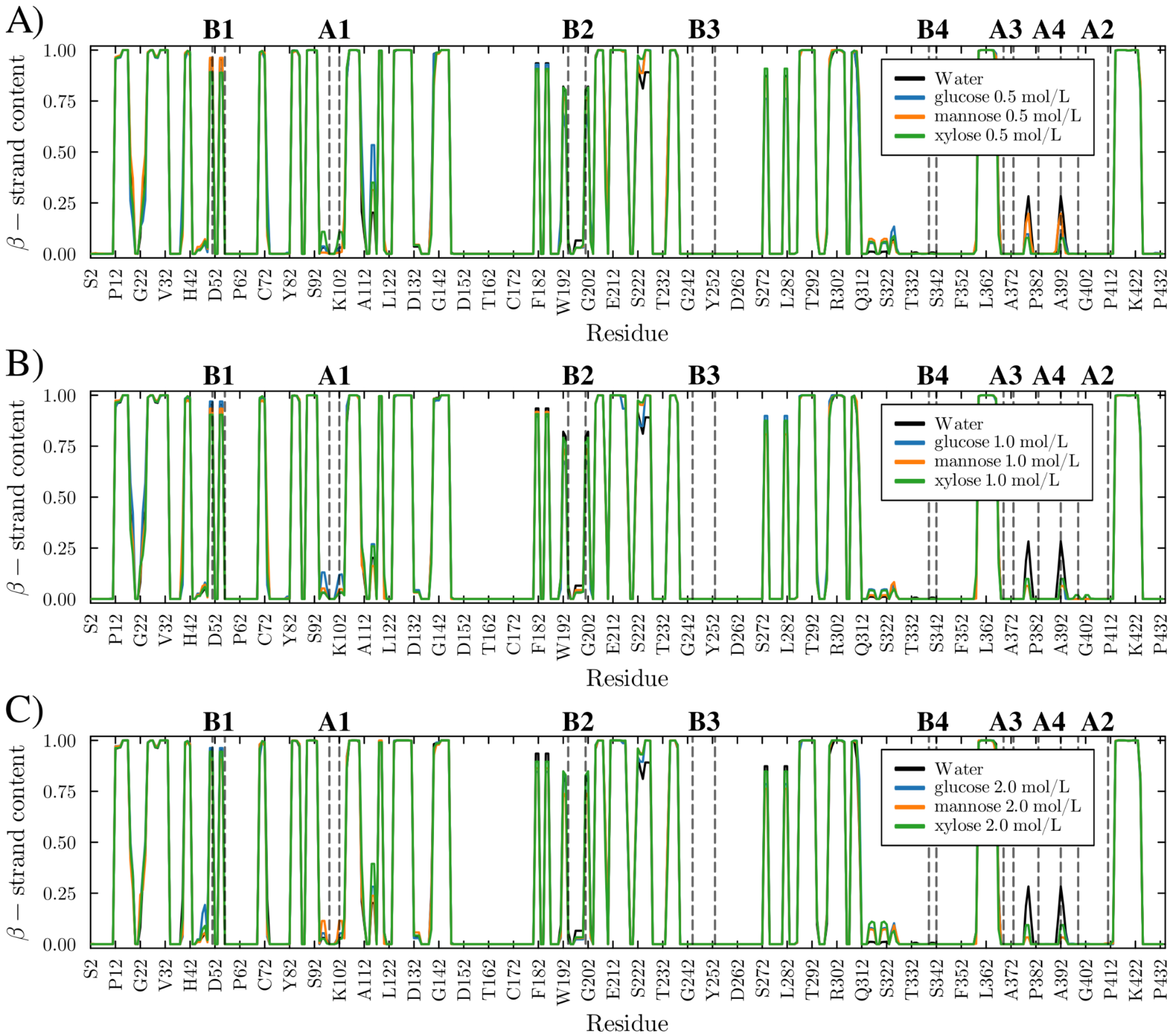

Figure S15. Average β-sheet content per residue for the Cel7A CD. Data represent the mean across five independent replicas. β-sheet profiles for the CD in water (black), β-D-glucose (blue), β-D-mannose (orange), and β-D-xylose (green) are shown for comparison. Panels A)-C) display the results for sugar concentrations of 0.5 M, 1.0 M, and 2.0 M, respectively. Gray dotted lines delimit the loop regions.

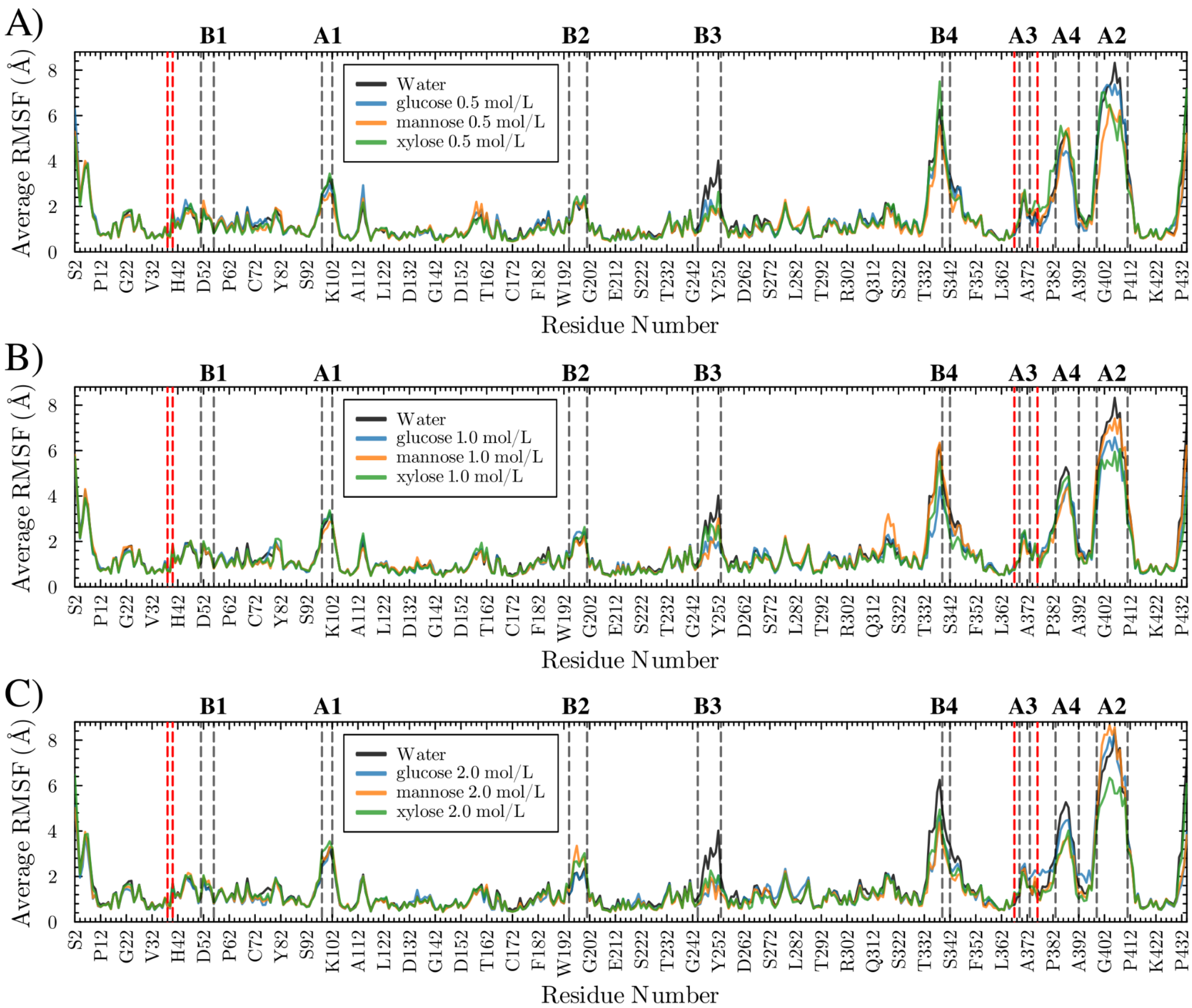

Figure S16. Average Root Mean Square Fluctuation (RMSF) per residue for the Cel7A CD. Data represent the mean across five independent replicas. WASA profiles for the CD in water (black), β-D-glucose (blue), β-D-mannose (orange), and β-D-xylose (green) are shown for comparison. Panels A)-C) display the results for sugar concentrations of 0.5 M, 1.0 M, and 2.0 M, respectively. Gray dotted lines delimit the loop regions, while red dotted lines indicate the positions of W38, W40, W367, and W376.

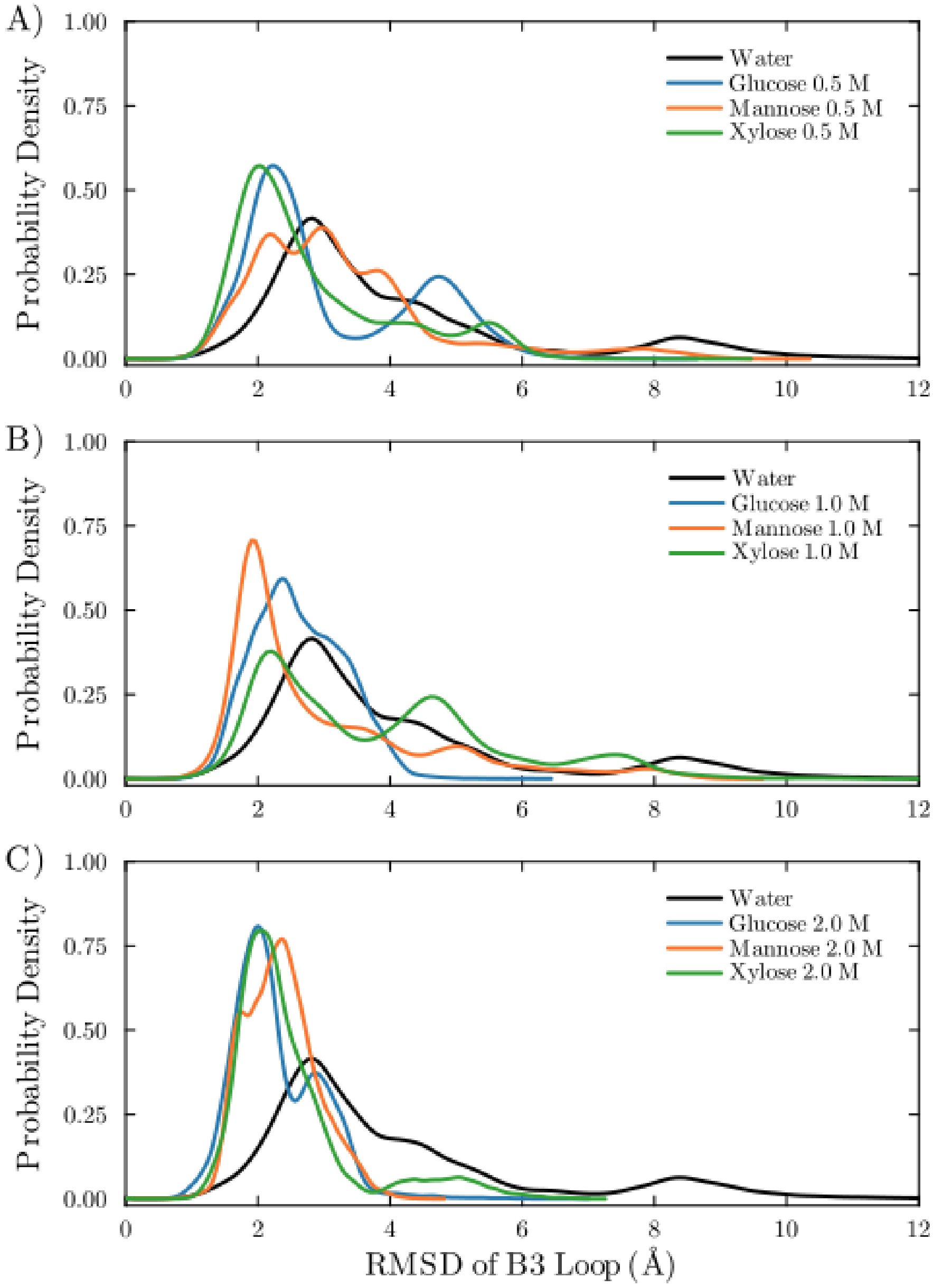

Figure S17. Kernel Density Estimation (KDE) of the B3 loop RMSD. Distributions are shown for pure water (black) and β-D-glucose (blue), β-D-mannose (orange), and β-D-xylose (green) solutions at A) 0.5 M, B) 1.0 M, and C) 2.0 M.

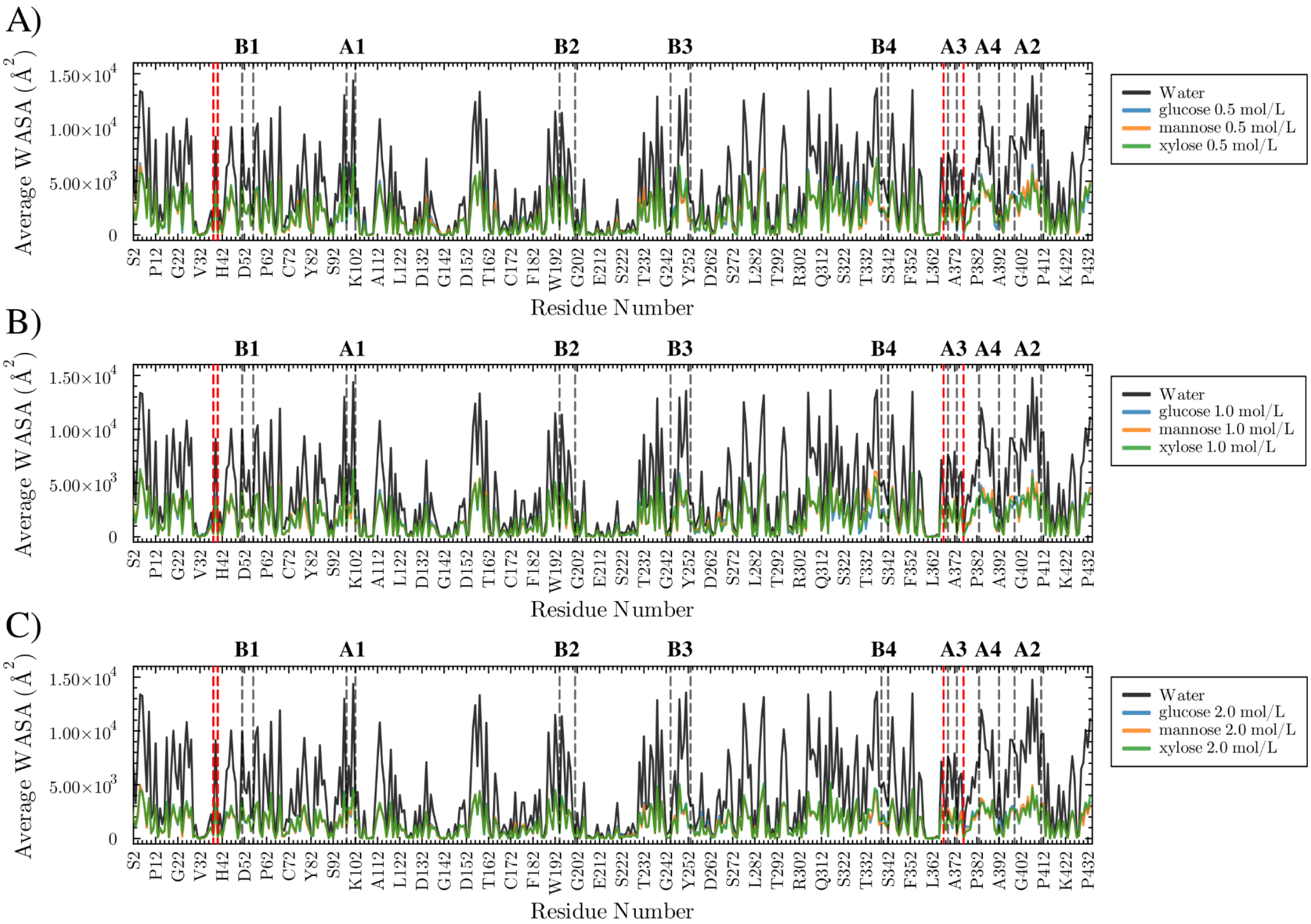

Figure S18. Average Water Accessible Surface Area (WASA) per residue for the Cel7A CD. Data represent the mean across five independent replicas. WASA profiles for the CD in water (black), β-D-glucose (blue), β-D-mannose (orange), and β-D-xylose (green) are shown for comparison. Panels A)-C) display the results for sugar concentrations of 0.5 M, 1.0 M, and 2.0 M, respectively. Gray dotted lines delimit the loop regions, while red dotted lines indicate the positions of the W38, W40, W367, and W376.

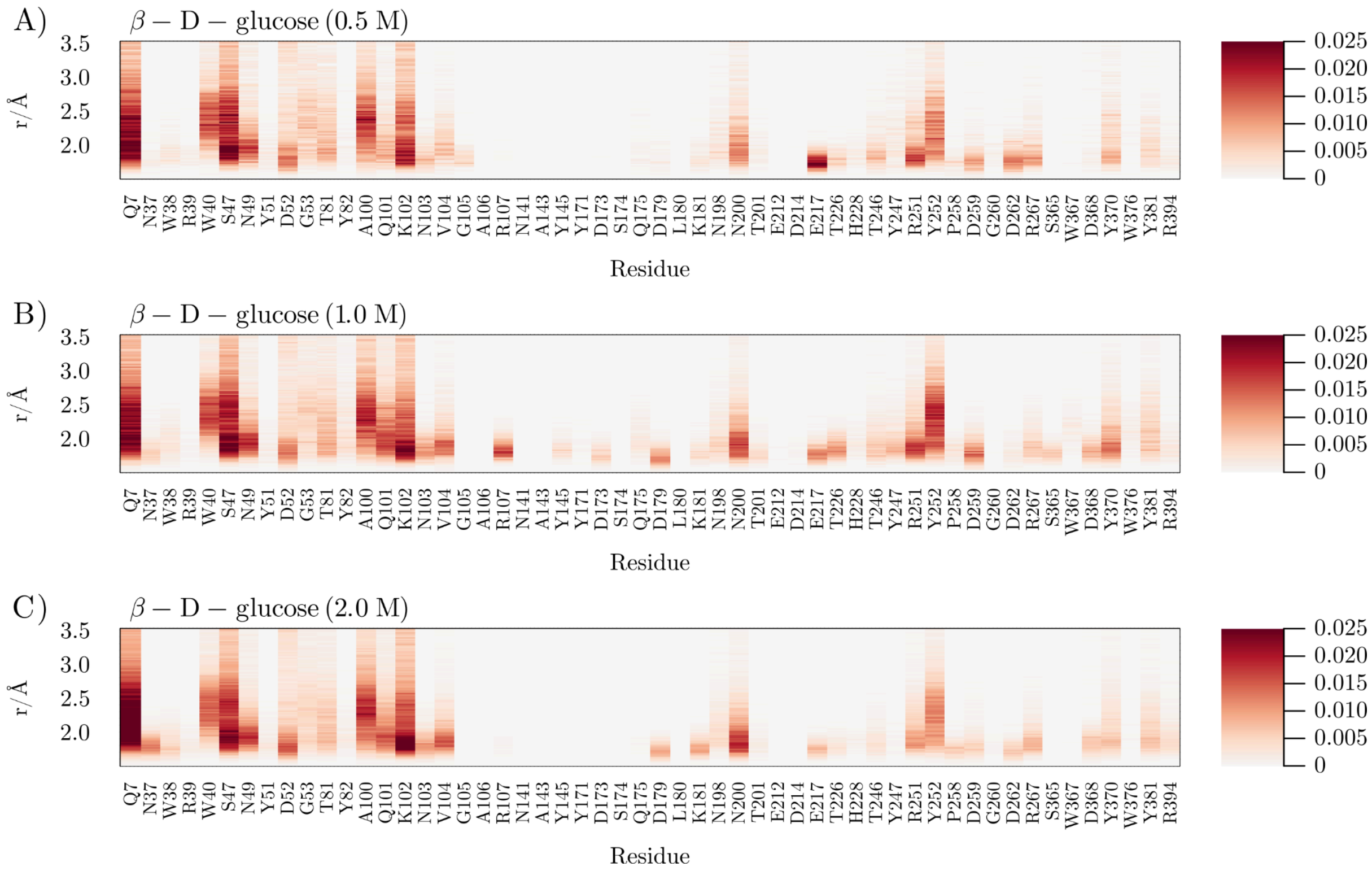

Figure S19.1. Contributions of tunnel residues (within 5 Å of the substrate path) of the Cel7A CD to the β-D-glucose MDDF. Panels A)-C) display the MDDFs for three distinct β-D-glucose concentrations (0.5 M, 1.0 M, and 2.0 M). The color scale ranges from 0.0 (white) to 0.025 (red), where red regions indicate greater β-D-glucose density near specific CD residues.

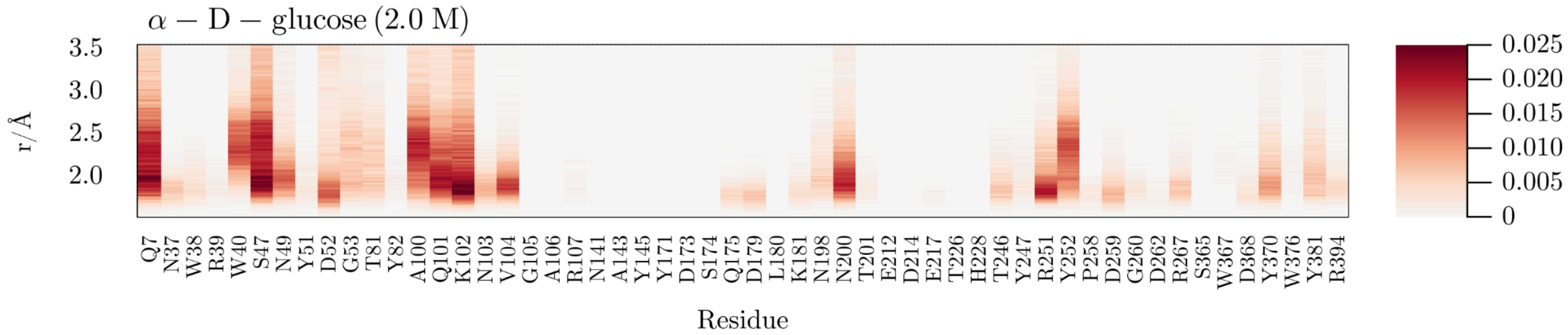

Figure S19.2. Contributions of tunnel residues (within 5 Å of the substrate path) of the Cel7A CD to the α-D-glucose MDDF. The color scale ranges from 0.0 (white) to 0.025 (red), where red regions indicate greater α-D-glucose density near specific CD residues.

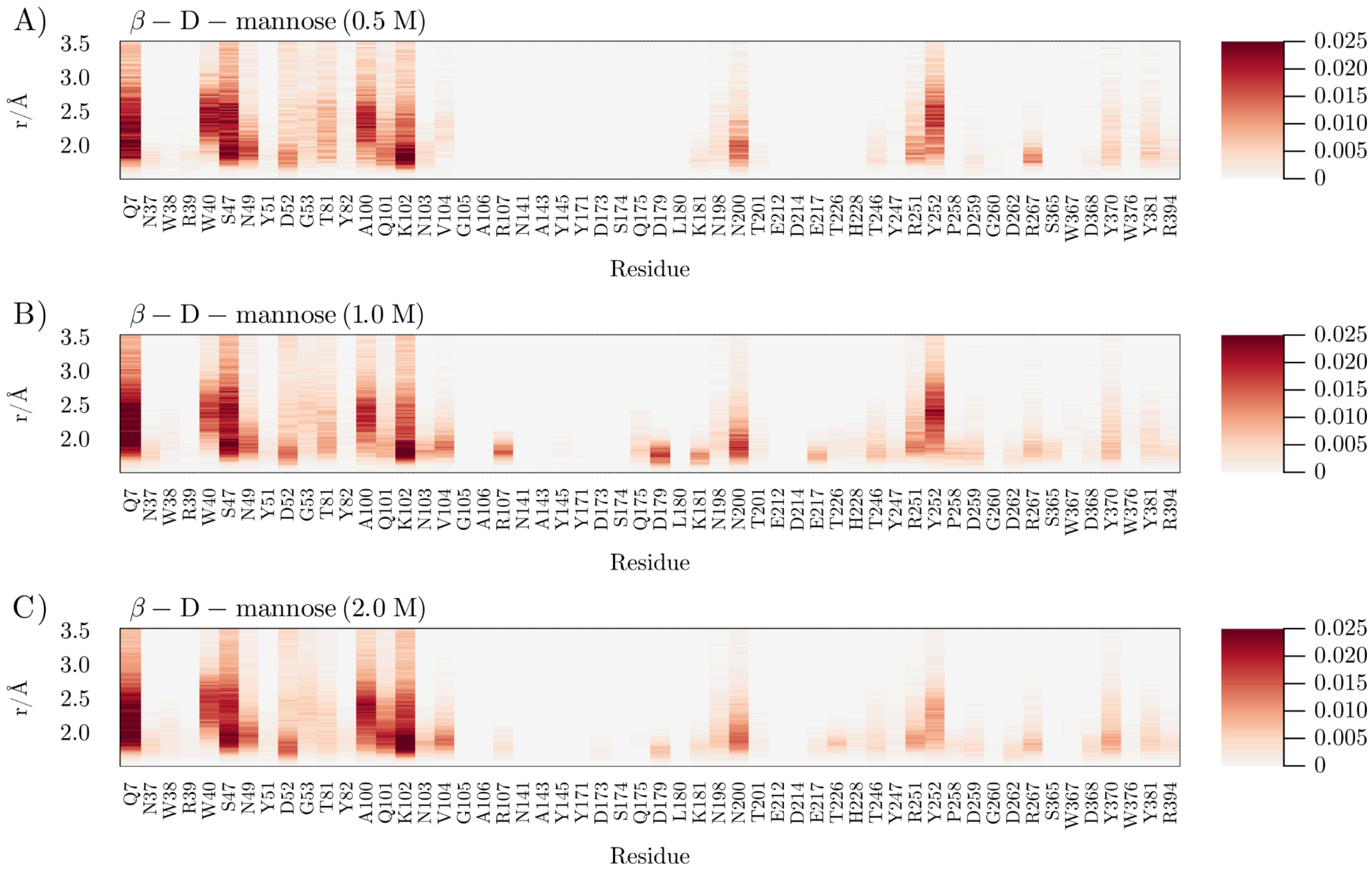

Figure S20.1. Contributions of tunnel residues (within 5 Å of the substrate path) of the Cel7A CD to the β-D-mannose MDDF. Panels A)-C) display the MDDFs for three distinct β-D-mannose concentrations (0.5 M, 1.0 M, and 2.0 M). The color scale ranges from 0.0 (white) to 0.025 (red), where red regions indicate greater β-D-mannose density near specific CD residues.

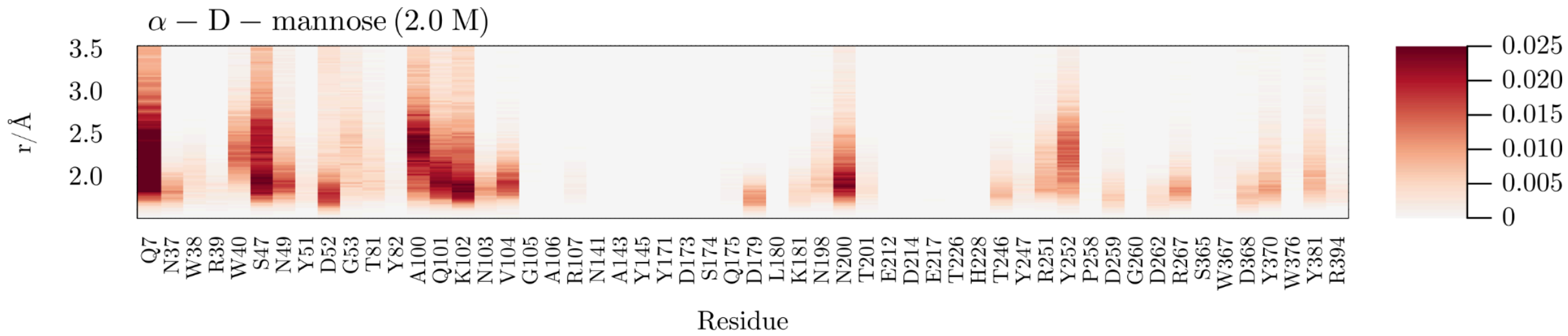

Figure S20.2. Contributions of tunnel residues (within 5 Å of the substrate path) of the Cel7A CD to the α-D-mannose MDDF. The color scale ranges from 0.0 (white) to 0.025 (red), where red regions indicate greater α-D-mannose density near specific CD residues.

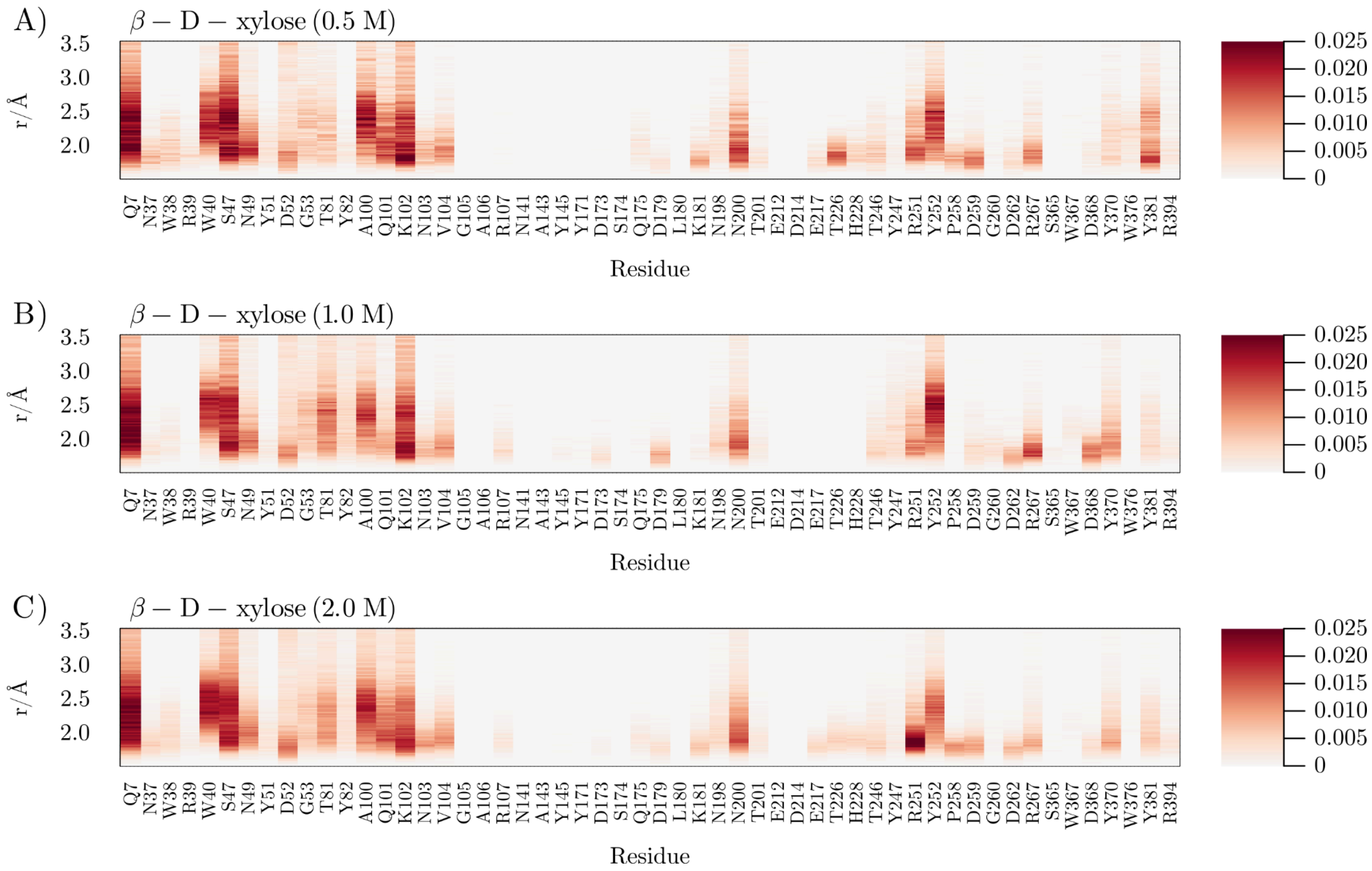

Figure S21.1. Contributions of tunnel residues (within 5 Å of the substrate path) of the Cel7A CD to the β-D-xylose MDDF. Panels A)-C) display the MDDFs for three distinct β-D-xylose concentrations (0.5 M, 1.0 M, and 2.0 M). The color scale ranges from 0.0 (white) to 0.025 (red), where red regions indicate greater β-D-xylose density near specific CD residues.

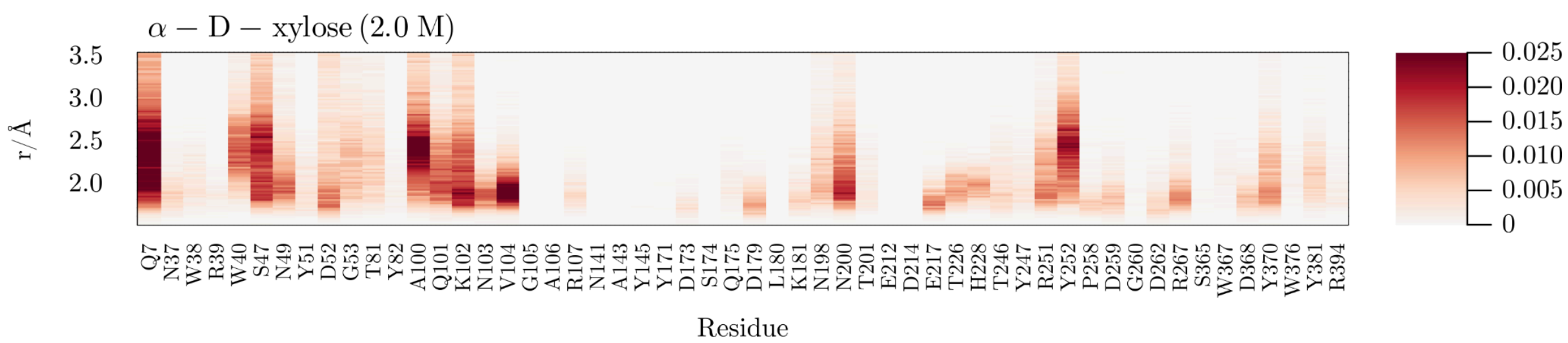

Figure S21.2. Contributions of tunnel residues (within 5 Å of the substrate path) of the Cel7A CD to the α-D-xylose MDDF. The color scale ranges from 0.0 (white) to 0.025 (red), where red regions indicate greater α-D-xylose density near specific CD residues.

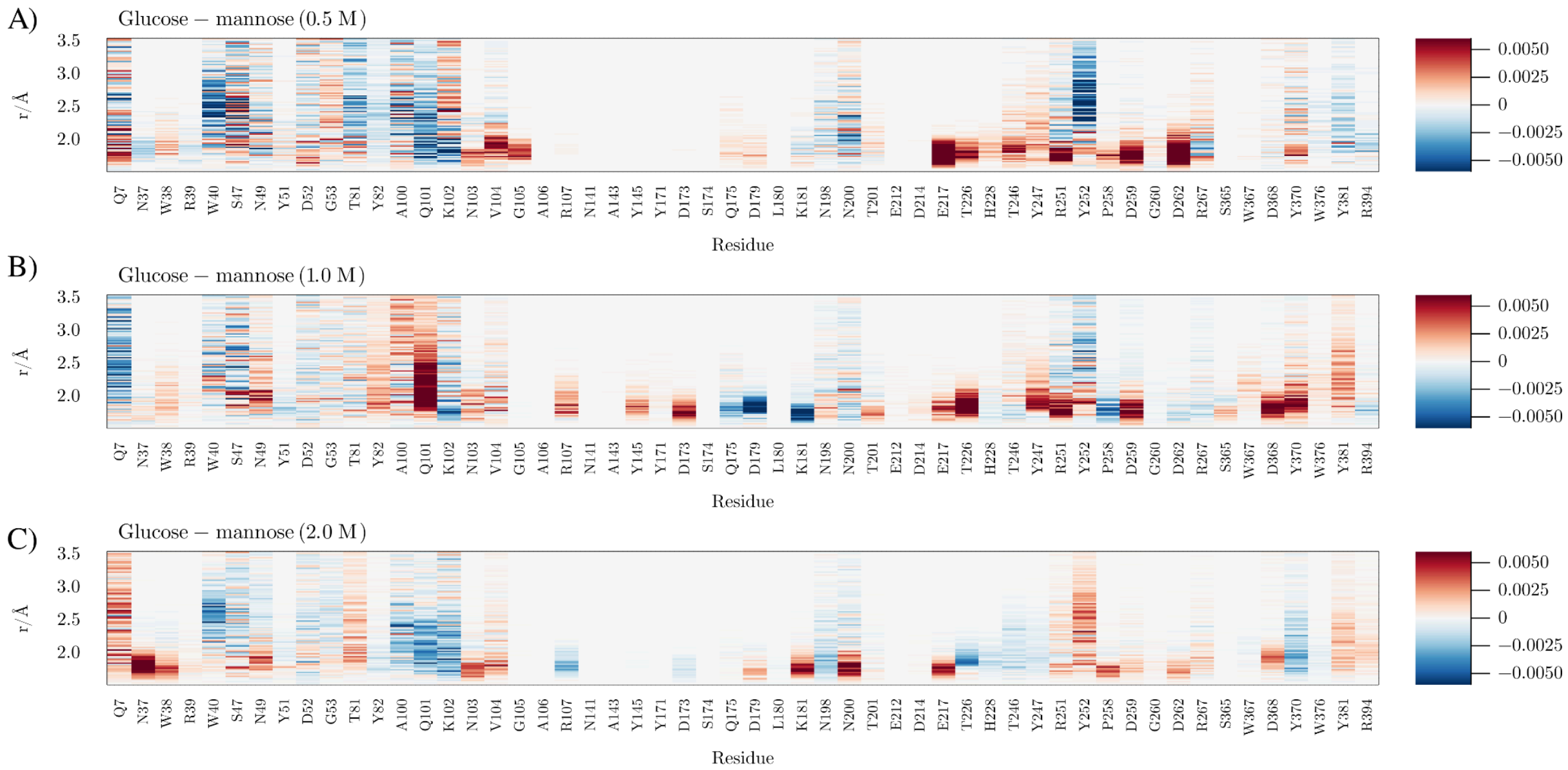

Figure S22.1. Differential β-D-glucose vs. β-D-mannose MDDF contributions for the Cel7A CD tunnel residues (within 5 Å of the substrate path). Panels A)-C) show the difference in residue-specific contributions at 0.5 M, 1.0 M, and 2.0 M. Red regions indicate higher β-D-glucose density, whereas blue regions represent a higher β-D-mannose density. White regions denote similar occupancy for both sugars.

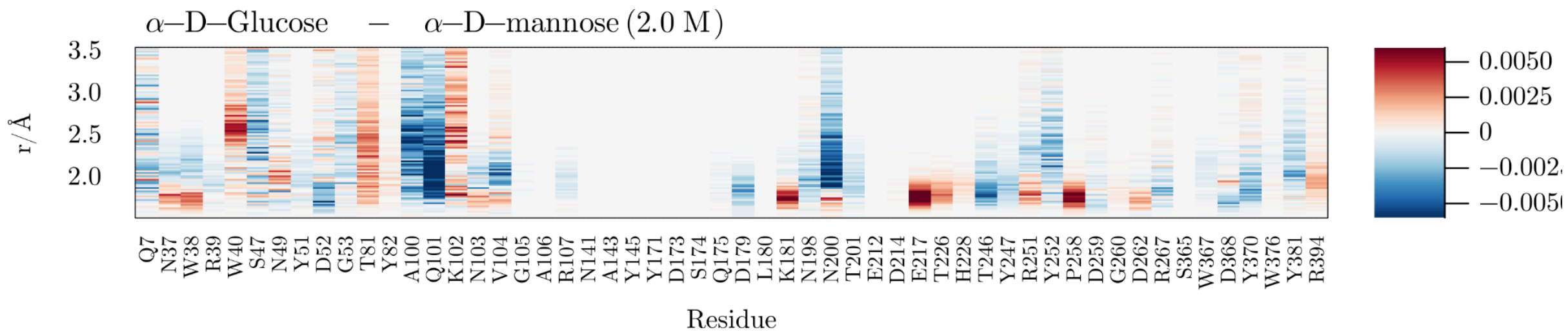

Figure S22.2. Differential α-D-glucose vs. α-D-mannose MDDF contributions for the Cel7A CD tunnel residues (within 5 Å of the substrate path). Red regions indicate higher α-D-glucose density, whereas blue regions represent a higher α-D-mannose density. White regions denote similar occupancy for both sugars.

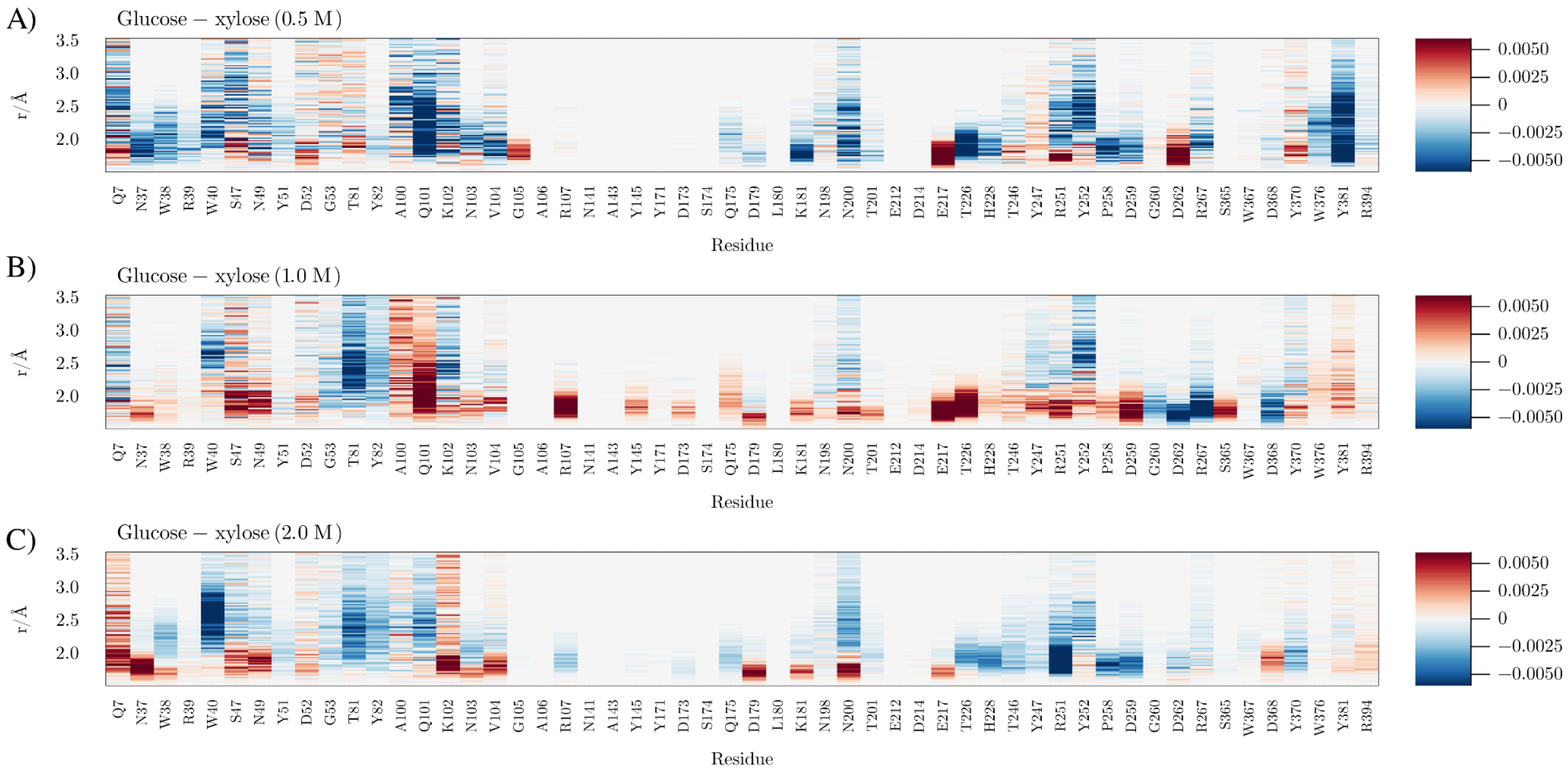

Figure S23.1. Differential β-D-glucose vs. β-D-xylose MDDF contributions for the Cel7A CD tunnel residues (within 5 Å of the substrate path). Panels A)-C) show the difference in residue-specific contributions at 0.5 M, 1.0 M, and 2.0 M. Red regions indicate higher β-D-glucose density, whereas blue regions represent a higher β-D-xylose density. White regions denote similar occupancy for both sugars.

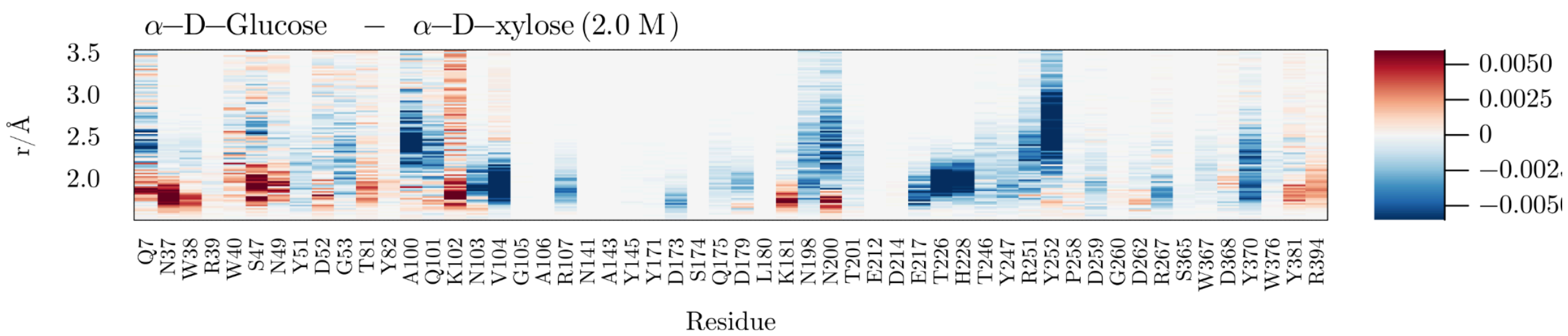

Figure S23.2. Differential α-D-glucose vs. α-D-xylose MDDF contributions for the Cel7A CD tunnel residues (within 5 Å of the substrate path). Red regions indicate higher α-D-glucose density, whereas blue regions represent a higher α-D-xylose density. White regions denote similar occupancy for both sugars.

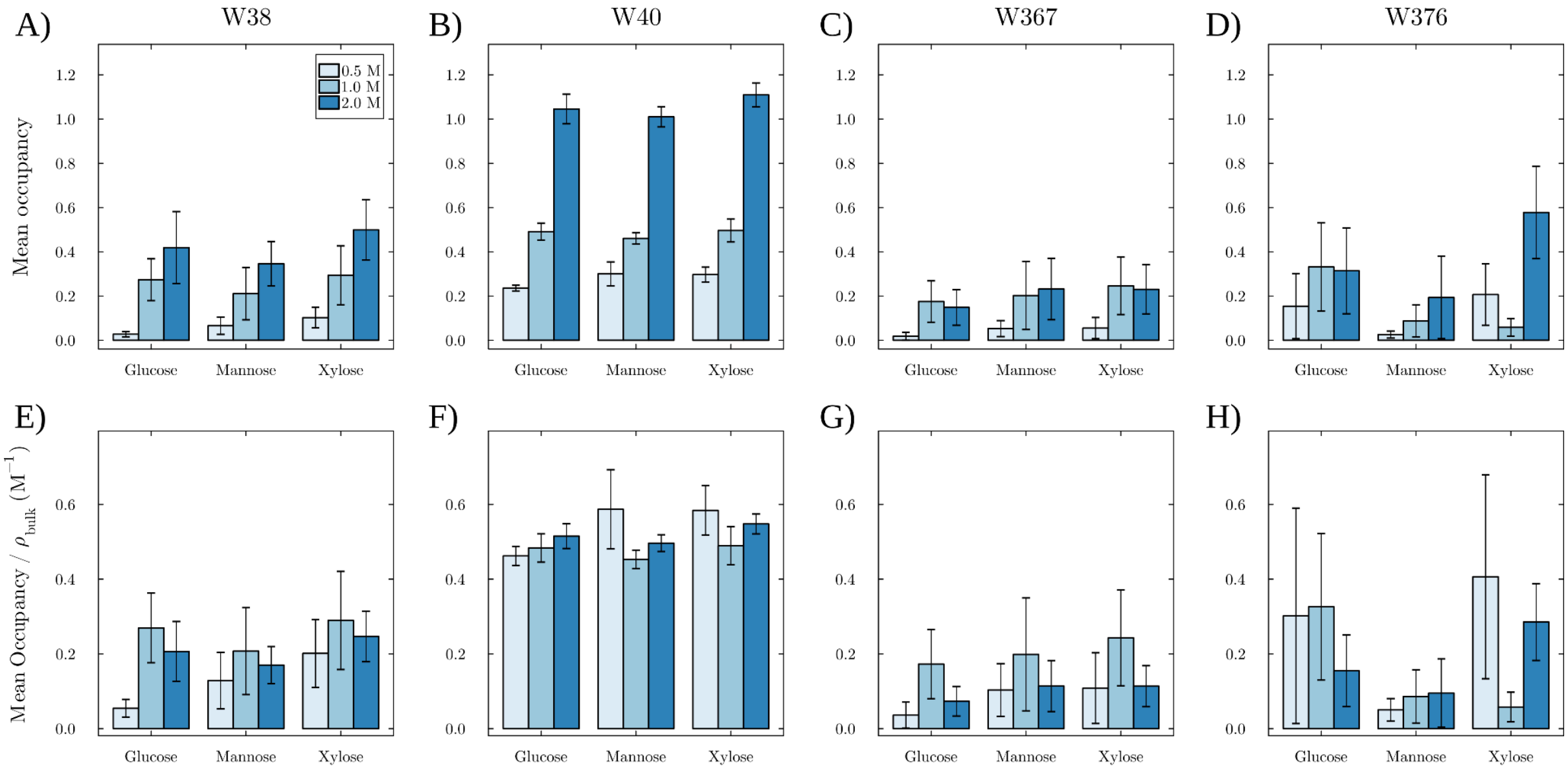

Figure S24. A)-D) Occupancy of W38, W40, W367, and W376 in the CD at 3.0 Å across sugars (β-D-glucose, β-D-mannose, and β-D-xylose) and concentrations (0.5, 1.0, and 2.0). E)-H) Normalized occupancy is defined as the occupancy divided by the average bulk density of the sugar in each system.
